# Transcriptomic Analysis of the Response of the *Dioryctria abietella* Larva Midgut to *Bacillus thuringiensis* 2913 Infection

**DOI:** 10.3390/ijms252010921

**Published:** 2024-10-10

**Authors:** Ruting Chen, Yutong Zhuang, Meiling Wang, Jia Yu, Defu Chi

**Affiliations:** Key Laboratory for Sustainable Forest Ecosystem Management of Ministry of Education, College of Forestry, Northeast Forestry University, Harbin 150040, China; crt1391403031@126.com (R.C.); zhuangyt0522@163.com (Y.Z.); 15043437530@163.com (M.W.); rainbest2013@126.com (J.Y.)

**Keywords:** *Dioryctria abietella*, *Bacillus thuringiensis*, activation of *Cry*/*Vip* toxin, receptor-protein binding, metabolic detoxification, immune defense response

## Abstract

*Dioryctria abietella* Denis Schiffermuller (Lepidoptera: Pyralidae) is an oligophagous pest that mainly damages Pinaceae plants. Here, we investigated the effects of the *Bacillus thuringiensis* 2913 strain (*Bt* 2913), which carries the *Cry*1Ac, *Cry*2Ab, and *Vip*3Aa genes, on the *D. abietella* midgut transcriptome at 6, 12, and 24 h after infection. In total, 7497 differentially expressed genes (DEGs) were identified from the midgut transcriptome of *D. abietella* larvae infected with *Bt* 2913. Among these DEGs, we identified genes possibly involved in *Bt* 2913-induced perforation of the larval midgut. For example, the DEGs included 67 genes encoding midgut proteases involved in *Cry*/*Vip* toxin activation, 74 genes encoding potential receptor proteins that bind to insecticidal proteins, and 19 genes encoding receptor NADH dehydrogenases that may bind to *Cry*1Ac. Among the three transcriptomes, 88 genes related to metabolic detoxification and 98 genes related to immune defense against *Bt* 2913 infection were identified. Interestingly, 145 genes related to the 60S ribosomal protein were among the DEGs identified in the three transcriptomes. Furthermore, we performed bioinformatic analysis of zonadhesin, GST, CYP450, and CarE in the *D. abietella* midgut to determine their possible associations with *Bt* 2913. On the basis of the results of this analysis, we speculated that trypsin and other serine proteases in the *D. abietella* larval midgut began to activate *Cry*/*Vip* prototoxin at 6 h to 12 h after *Bt* 2913 ingestion. At 12 h after *Bt* 2913 ingestion, chymotrypsin was potentially involved in degrading the active core fragment of *Vip*3Aa toxin, and the detoxification enzymes in the larvae contributed to the metabolic detoxification of the *Bt* toxin. The ABC transporter and several other receptor-protein-related genes were also downregulated to increase resistance to *Bt* 2913. However, the upregulation of 60S ribosomal protein and heat shock protein expression weakened the resistance of larvae to *Bt* 2913, thereby enhancing the expression of NADH dehydrogenase and other receptor proteins that are highly expressed in the larval midgut and bind to activating toxins, including *Cry*1Ac. At 24 h after *Bt* 2913 ingestion, many activated toxins were bound to receptor proteins such as APN in the larval midgut, resulting in membrane perforation. Here, we clarified the mechanism of *Bt* 2913 infection in *D. abietella* larvae, as well as the larval immune defense response to *Bt* 2913, which provides a theoretical basis for the subsequent control of *D. abietella* using *B. thuringiensis*.

## 1. Introduction

**Figure 1:**
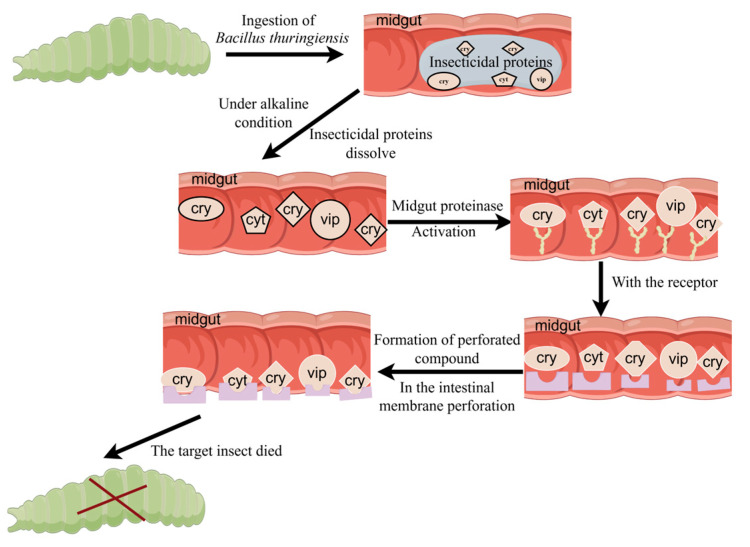
Insecticidal mechanism of *Bacillus thuringiensis* (produced by Figdraw https://www.figdraw.com/, 8 October 2024).

*Bacillus thuringiensis* (*Bt*) is an entomopathogenic bacterium. It produces insecticidal crystal proteins (ICPs), and vegetative insecticidal proteins (Vips) are pathogenic to a variety of insects, such as Lepidoptera and Coleoptera [1,2,3]. *Bt* Vips are produced during the vegetative growth phase; whereas, ICPs are formed upon spore release and are classified into the *Cry* and *Cyt* families [4,5]. Insecticidal proteins have specific toxicity in Lepidoptera pests, and the insecticidal proteins mainly include *Cry*1, *Cry*2, *Cry*9, and *Vip*3, which have distinct activities [1,2]. *Cry* and *Vip* toxins are among the insecticidal protein complex of *Bt* and are present in the form of prototoxins. When the target insect ingests *Bt*, the insecticidal protein complex dissolves and releases the prototoxin in the insect midgut under alkaline conditions. The prototoxins are activated by trypsin and other midgut proteases to produce the corresponding activated toxins, which bind to receptors on the midgut surface. The perforating complex is further formed and inserted into the midgut cell membrane to form a pore, thereby disturbing the osmotic balance of the cell and causing the death of the target insect [1,6,7,8] (Figure 1).

Korean pine (*Pinus koraiensis* Sieb. et Zucc) is distributed in northeastern China; its pine nuts have high nutritional value and are very important and valuable to the natural heritage of the area [9]. *Dioryctria abietella* Denis Schiffermuller (Lepidoptera: Pyralidae), an oligophagous pest, is distributed in northeastern China, Tibet, Yunnan, and other regions [10,11,12]. Because *D. abietella* larvae often feed on the cones of Pinaceae plants such as *P. koraiensis*, the cone development is poor, and the pine seed yield is reduced, which is extremely destructive to coniferous forests, such as Korean pine forests [13]. The continuous application of pesticides causes soil pollution and changes the soil microbial community [14,15]. Therefore, alternative pesticides for the control of *D. abietella* are needed to reduce adverse impacts on the environment. However, Zhang et al. reported that endogenous serine protease inhibitors in insects can block the activation of protoxins by serine proteases, thereby reducing the virulence of *Bt* [16]. Detoxification enzymes, such as carboxylesterases (CarEs), cytochrome P450s (CYP450s), glutathione S-transferases (GSTs), and uridine diphosphate (UDP)−glycosyltransferases (UGTs) in insects are often involved in xenobiotic metabolism [17,18,19]. Insects are, thus, resistant to drugs [20].

In our prior investigation, the lethality of *Bt* 2913 on fifth instar larvae of *D. abietella* was determined to be 61.67 ± 6.24% at a concentration of 1 × 10^8^ CFU/mL. Additionally, the larvae exhibited robust protective and detoxification enzyme activities [21]. We hypothesize that this observation may be attributed to the enhanced immune defense mechanisms of *D. abietella* larvae against *Bt* 2913. Therefore, we used transcriptomics to study the changes in serine protease, receptor protein, detoxification enzyme, and serine protease inhibitor gene expression in the midgut of *D. abietella* at different time points after the ingestion of *B. thuringiensis* 2913 (*Bt* 2913). Furthermore, the mechanism by which *Bt* affects *D. abietella* and the mechanism of the *D. abietella* immune response to *Bt* stress were further analyzed. These results lay the foundation for the subsequent biological control of *D. abietella* by *Bt*.

## 2. Results

### 2.1. Identification of the Genotype of Bt 2913 and Its Parasporal Crystal Metabolism

**Figure 2:**
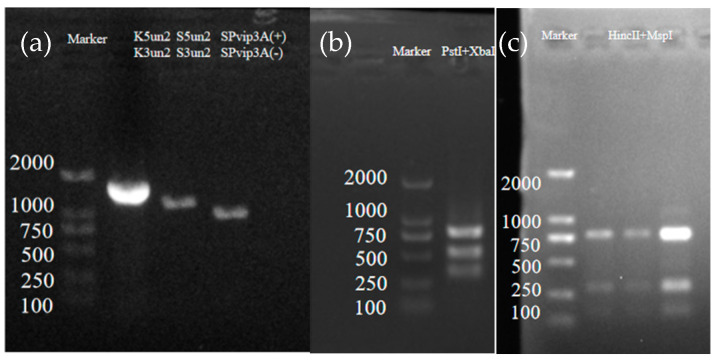
PCR amplification results of the *Cry*/*Vip* genes and PCR-RFLP identification fingerprints. (**a**) From left to right, the PCR amplification results of Marker, K5un2/K3un2, S5un2/S3un2, and SPvip3A(+)/SPvip3A(−) are shown; (**b**) PCR-RFLP patterns of PstI+XbaI double-digestion primer K5un2/K3un2; (**c**) PCR-RFLP patterns of HincII+MspI double-digestion primer S5un2/S3un2.

The *Cry*1-9 gene of the *Bt* 2913 strain was identified via PCR-RFLP, and *Vip*3Aa-specific primers were used to identify *Vip*3Aa (Tables S1 and S2). Among the targets of the primers used for identification, only the *Cry*1, *Cry*2, and *Vip*3Aa fragments were amplified (Figure 2a). The PCR-RFLP patterns of the PstI+XbaI double-digestion primer K5un2/K3un2 amplification products were 322 bp, 801 bp, and 518 bp, respectively (Figure 2b). The PCR-RFLP patterns of the HincII+MspI double-digestion primer S5un2/S3un2 amplification products were 791, 297, and 143 bp, respectively (Figure 2c). The sequences of K5un2/K3un2, S5un2/S3un2, and SPVip3A(+)/SPVip3A(−) were analyzed via NCBI BLAST. The results revealed that the K5un2/K3un2, S5un2/S3un2, and SPVip3A(+)/SPVip3A(−) amplification products had the highest homology with *Cry*1Ac (MK184463.1, 99.94%), *Cry*2Ab (MK184463.1, 99.94%), and *Vip*3Aa (KY780302.1, 99.89%), respectively. Therefore, the *Bt* 2913 strain contains the *Cry*1Ac, *Cry*2Ab, and *Vip*3Aa genes. Because the *Cry*1Ac (PQ246116), *Cry*2Ab (PQ246117), and *Vip*3Aa (PQ246118) proteins all have toxic effects on Lepidoptera insects, *Bt* 2913 can be used for the control of *D. abietella*. 

**Figure 3:**
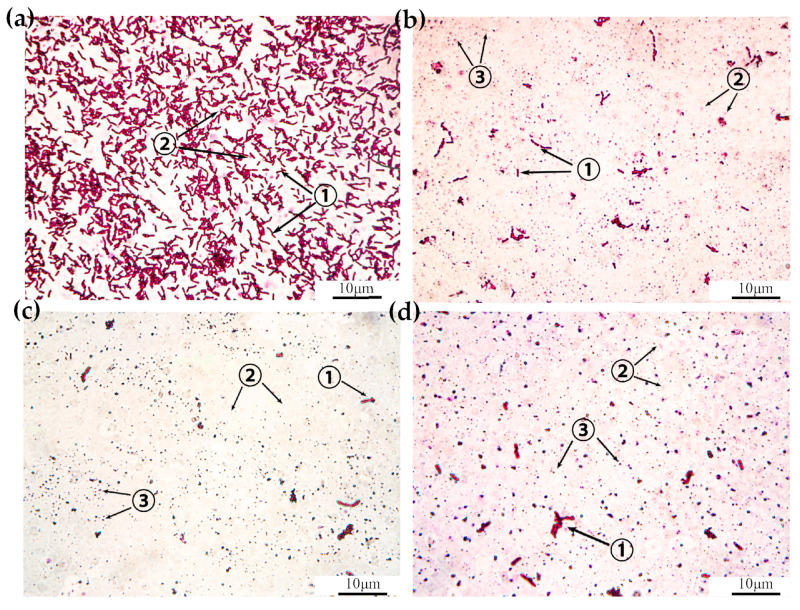
Parasporal crystal metabolism at 30 °C and 200 r/min (observed with a 40× oil lens). (**a**) Culture for 24 h; (**b**) culture for 26 h; (**c**) culture for 30 h; (**d**) culture for 48 h. The red rod shape in the form shown in ① represents the *Bt* 2913 strain; the light red oval shape in the form shown in ② represents the spore; and the dark irregular crystals in the form shown in ③ represent parasporal crystals.

Staining microscopy was used to examine the *Bt* 2913 bacterial culture at 30 °C and 200 r/min in a constant-temperature shaker for 24 h. The shape of the thallus was complete, and the number of spores was large. A small number of spores were produced near the thallus, and there was no parasporal crystal metabolism (Figure 3a). At 26 h of culture, many spores were produced, which were exfoliated, and a small number of parasporal crystals were metabolized (Figure 3b). After 30 h of culture, the parasporal crystals began to be metabolized in large quantities (Figure 3c). However, after 48 h of culture, the parasporal crystals exhibited a sluggish metabolism (Figure 3d). Therefore, a bacterial solution of *Bt* 2913, which had been cultured at 30 °C and 200 r/min in a constant-temperature shaker for 30 h, was used to treat *D. abietella*.

### 2.2. Transcriptome in the Midgut Tissue of D. abietella Larvae in the Context of Bt 2913 Infection

Transcriptome sequencing was used to investigate the response of the midgut tissue of *D. abietella* larvae to *Bt* 2913 infection. The number of raw reads in the 6 h (CS), 12 h (CT), and 24 h (CF) control libraries without *Bt* 2913 infection and the 6 h (BS), 12 h (BT), and 24 h (BF) treated libraries with *Bt* 2913 infection ranged from 46,659,924 to 136,336,758. The number of clean reads ranged from 37,809,978 to 130,172,720. The number of bases, average read length, base ratio, and base quality of the raw and clean RNA sequencing data are shown in Table S3. Bowtie2 was used to compare the quality control sequence with the reference sequence obtained via Trinity splicing, and the total mapped range was 79.31% to 84.78%. A total of 365,596 transcripts and 192,963 unigenes were obtained via de novo assembly, of which 42,403 transcripts and 17,614 unigenes had sequences greater than or equal to 1000 bp. The N50 lengths of the transcripts and unigenes were 785 bp and 633 bp, respectively (Table S4).

A total of 192,963 unigenes were annotated in the CDD, PFAM, KEGG, KOG, GO, NR, and NT databases, of which 67,532 unigenes were annotated in the NR database. A total of 21,923 and 37,946 unigenes were annotated in the KEGG and GO databases, respectively, and 2814 unigenes were annotated in all the databases (Table S5).

A comparison of the transcript sequences of the midgut tissues of *D. abietella* larvae with sequences in the NR database revealed that the transcript sequences were generally similar to sequences in the insect genome. According to NR database homologous species classification, 26% of the NR database annotated unigenes were homologous to Amyelois transitella sequences, and 5% of the unigenes were homologous to Lucilia cuprina sequences. In total, 3% of the unigenes were homologous to Papilio xuthus, L. sericata and Galleria mellonella sequences, and the proportion of homology with other insects of the remaining sequences is shown in Figure S1. 

The GO classification of the resulting genes resulted in 37,946 unigenes in 68 GO terms. There were 20, 26, and 22 GO terms associated with the biological process (BP), cellular component (CC), and molecular function (MF), respectively. In the BP category, 31,966, 26,444, and 20,881 unigenes were annotated as “cellular process”, “metabolic process”, and “biological regulation”, respectively. In the CC category, 32,885, 25,156, 14,588, and 14,542 unigenes were annotated as “cell”, “organelle”, “membrane”, and “protein-containing complex”, respectively. The terms “binding” and “catalytic activity” were the top two terms in the MF category, with 21,759 and 16,404 unigenes, respectively (Figure S2).

At present, KEGG has established a complete KEGG Orthology (KO) annotation system, which links all KEGG annotation systems together. After the KO annotation of the obtained genes, we performed a KEGG metabolic pathway classification of 21,923 unigenes, according to the connection between the KOs and pathways, and a total of 34 metabolic pathways were identified. The top 7 metabolic pathways were “signal transduction”, “translation”, “carbohydrate metabolism”, “overview”, “folding sorting and degradation”, “energy metabolism”, and “transport and catabolism”, which included 3806, 3321, 2991, 2365, 2335, 2038, and 2022 unigenes, respectively (Figure S3).

A total of 27,976 unigenes were annotated in the KOG database and classified into 25 groups, according to the KOG group. Among them, 3231 and 3164 unigenes were classified into the groups “general function prediction only” and “posttranslational modification, protein turnover, chaperones”, respectively. A total of 2889 and 2790 unigenes were classified into the groups “signal transduction mechanisms” and “translation, ribosomal structure and biogenesis”, respectively (Figure S4).

### 2.3. Analysis of Differentially Expressed Genes (DEGs) in the Midgut Tissues of Bt 2913-Treated D. abietella Larvae at Different Time Points

**Figure 4:**
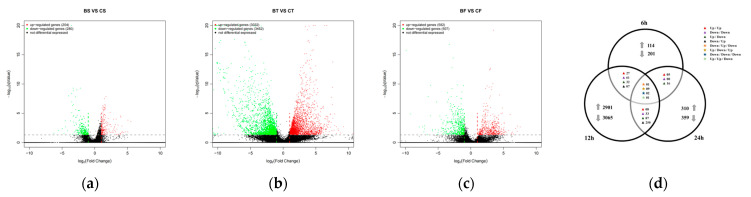
DEGs in the midgut tissues of *Bt* 2913-treated *D. abietella* larvae at different time points. (**a**–**c**) DEGs in the midgut of the *D. abietella* larvae fed on *Bt* 2913 for 6 h, 12 h, and 24 h, respectively; (**d**) Venn diagram showing the number of *D. abietella* genes that were differentially expressed (upregulated or downregulated) at only 6 h, only 12 h, or only 24 h or at multiple time points after treatment with *Bt* 2913.

To investigate the mechanism by which *D. abietella* larvae responded to *Bt* 2913 at different time points, we compared the midguts of larvae infected with *Bt* 2913 for 6 h, 12 h, and 24 h with those of their corresponding controls. DESeq2 was used to analyze the differences in gene expression in each group. A total of 7497 DEGs were identified among the 6 h, 12 h, and 24 h sample transcripts (Table S6). A total of 204 upregulated genes and 280 downregulated genes were identified when *D. abietella* larvae were infected with *Bt* 2913 for 6 h (Figure 4a). When the larvae were infected with *Bt* 2913 for 12 h, a total of 6484 DEGs were identified, of which 3032 were upregulated and 3452 were downregulated (Figure 4b). After 24 h of infection with *Bt* 2913, 582 upregulated genes and 507 downregulated genes were identified (Figure 4c). In addition, 13 genes were identified at all three time points, among which one gene was downregulated at 6 h and 24 h and upregulated at 12 h; 9 genes were upregulated at 6 h and 24 h and downregulated at 12 h; 2 genes were downregulated at all three time points; 1 gene was upregulated at 6 h and 12 h and downregulated at 24 h; 27 genes were upregulated, and 61 genes were downregulated at 6 h and 12 h; 32 genes were upregulated at 6 h and downregulated at 12 h; and 7 genes were downregulated at 6 h and upregulated at 12 h. Among the 12 h and 24 h sample DEGs, 8 genes were upregulated, and 33 genes were downregulated at both time points; 87 genes were upregulated at 12 h and downregulated at 24 h; and 250 genes were downregulated at 12 h and upregulated at 24 h. Among the 6 h and 24 h sample DEGs, 5 genes were upregulated, and 8 genes were downregulated at both time points; 16 genes were upregulated at 6 h and downregulated at 24 h (Figure 4d).

The results of the KEGG pathway enrichment analysis and GO functional enrichment analysis of larvae fed on *Bt* 2913 at different time points are shown in Figure S5. 

### 2.4. Identification of Genes Associated with the Response to Bt 2913 Infection in the Midgut Tissue of D. abietella Larvae

**Figure 5:**
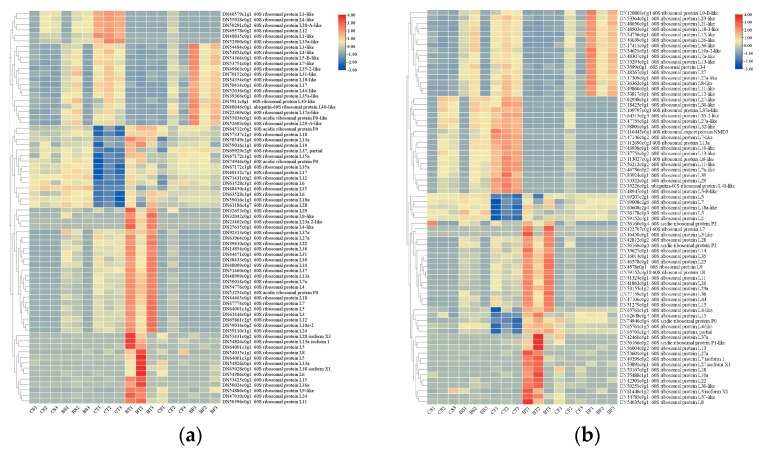
(**a**,**b**) Heatmaps of 60S ribosomal-protein-related DEGs in the midgut transcriptome at different periods after *D. abietella* was fed *Bt* 2913 (log2^(TPM+1)^ normalization was applied).

**Figure 6:**
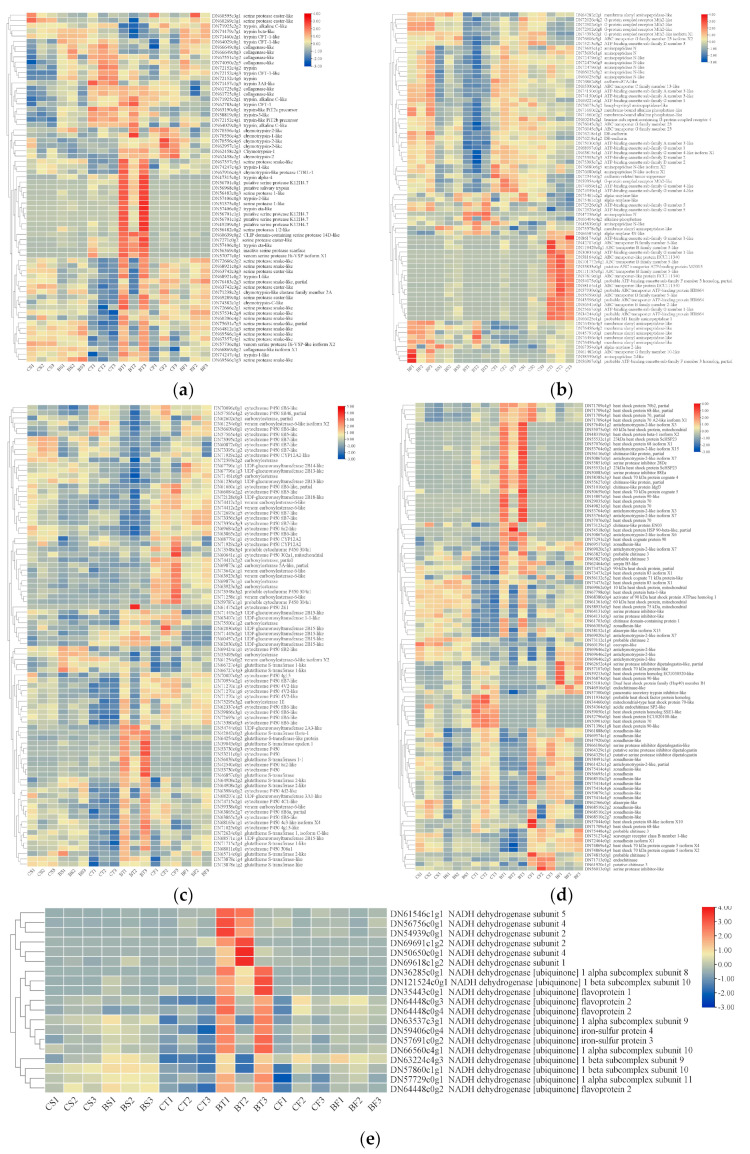
Heatmaps of DEGs in the midgut transcriptome at different time points after *D. abietella* was fed *Bt* 2913 (log2^(TPM+1)^ normalization was chosen). (**a**) Midgut proteases involved in *Bt* activation toxin; (**b**) potential *Bt* toxin receptor proteins; (**c**) metabolic detoxification genes; (**d**) genes associated with the immune defense response; (**e**) NADH-dehydrogenase-related genes.

According to the NR annotations of the DEGs at the three time points, there were a high number of genes related to the ribosomal protein, including 145 genes related to the 60S ribosomal protein (Figure 5a,b). According to the infection mechanism of *Bt*, 67 midgut protease genes that may be involved in *Cry*/*Vip* toxin activation (Figure 6a), 74 potential receptor protein genes that can bind to insecticidal proteins (Figure 6b), and 88 genes related to metabolic detoxification were identified from the midgut transcriptome at 6 h, 12 h, and 24 h after feeding on *Bt* 2913 (Figure 6c). In addition, 98 genes were involved in the insect’s immune defense (Figure 6d). Moreover, 19 genes related to NADH dehydrogenase were upregulated at the 12 h time point; whereas, no significant differences were detected at 6 h and 24 h (Figure 6e).

#### 2.4.1. Expression of Midgut Proteases Involved in Bt Toxin Activation

Among the midgut protease genes involved in *Bt* toxin activation (Figure 6a), a total of 14 DEGs were identified after *D. abietella* feeding on *Bt* 2913 for 6 h, among which the genes encoding 9 trypsins and 3 collagenases were upregulated, and the genes encoding 2 serine proteases were downregulated. A total of 49 DEGs were detected when *D. abietella* was fed on *Bt* 2913 for 12 h, among which the genes encoding 9 trypsin-related genes were upregulated, the genes encoding 4 trypsin-related genes were downregulated, the genes encoding 6 chymotrypsins and 27 serine proteases were upregulated, and 1 collagenase-encoding gene was upregulated; the genes encoding 2 collagenases were downregulated, 1 of which was the same gene as the upregulated collagenase gene. At 24 h after feeding on *Bt* 2913, the genes encoding 2 trypsins were identical to the downregulated genes encoding trypsins at 12 h, 1 upregulated and the other downregulated; 2 genes encoding trypsins were identical to the upregulated genes encoding trypsins at 6 h, 1 trypsin-encoding gene was upregulated, and the other was downregulated; the genes encoding 2 collagenase and 6 chymotrypsins were downregulated, and the genes encoding 3 chymotrypsins were identical to the upregulated genes encoding chymotrypsins at 12 h.

#### 2.4.2. Expression of Potential Receptor Proteins for Bt Toxin

Among the potential *Bt* toxin receptor protein genes screened (Figure 6b), 2 DEGs encoding alpha-amylases were upregulated in the midgut of larvae fed on *Bt* 2913 for 6 h and 12 h. In addition, 1 of the DEGs at 6 h was downregulated at 24 h in the midgut. In the midgut of larvae infected with *Bt* 2913 for 12 h, there were no DEGs related to G-protein-coupled receptors; whereas, 2 downregulated genes and 4 upregulated genes were found in the transcripts of the 6 h and 24 h groups, respectively. In the midgut transcriptome at three time points, only 3 alkaline phosphatases were expressed among the DEGs at 12 h, of which 2 were downregulated, and 1 was upregulated. A total of 4 cadherins were downregulated at 12 h and 24 h, but none were differentially expressed at 6 h. A total of 21 genes related to aminopeptidase were found in each group of DEGs, and 2 were differentially expressed at 6 h, of which 1 membrane alanyl aminopeptidase gene was upregulated. A total of 10 aminopeptidase N-encoding genes were detected among the 12 h DEGs, and only 1 such gene was upregulated. In addition, 1 M1 family aminopeptidase-related gene was downregulated at 6 h. A total of 8 DEGs were identified among the 24 h DEGs: only 1 was annotated via the NR database as an aminopeptidase N and downregulated, and the genes encoding the other 6 membrane alanyl aminopeptidases and 1 aminopeptidase were upregulated. In addition, we found that almost all the aminopeptidase N-encoding genes screened were downregulated, but all the membrane alanyl aminopeptidase-encoding genes were upregulated. A total of 36 ABC transporter-related DEGs were identified, of which 1 was downregulated at 6 h. There were 2 upregulated ABC transporter-related genes and 30 downregulated ABC transporter-related genes at 12 h. There were 2 upregulated and downregulated ABC transporter-related genes at 24 h, and 1 upregulated ABC transporter-related gene was downregulated at 12 h.

#### 2.4.3. Expression of Metabolic Detoxification Genes and 60S Ribosomal Proteins

After the plants were fed *Bt* 2913, 43 cytochrome P450 genes were found among the selected genes involved in metabolic detoxification (Figure 6c). Among these genes, 1 upregulated gene and 2 downregulated genes were identified after feeding on *Bt* 2913 for 6 h, and the downregulated genes were still downregulated after feeding for 12 h; 14 cytochrome P450 genes were downregulated, and 21 were upregulated, when the larvae were fed on *Bt* 2913 for 12 h. A total of 8 downregulated cytochrome P450 genes were detected after the plants were fed *Bt* 2913 for 24 h, and 1 gene was upregulated at 12 h. Through DEG screening of UDP-glucuronosyltransferase (UGT), it was found that the trend of UGT expression at different time points was essentially the same as that of cytochrome P450. There were 2 downregulated UGT genes at 6 h and 4 upregulated genes and 1 downregulated gene at 12 h. There were 6 downregulated genes and 2 upregulated genes at 24 h, and there was 1 UGT gene differentially expressed at 6 h, 12 h, and 24 h, which was downregulated, upregulated, and downregulated, respectively. There were 14 glutathione S-transferase (GST) differentially expressed genes that were upregulated at 6 h and 12 h, and no such DEGs were detected at 24 h. There were 3 downregulated and 2 upregulated carboxylesterase genes at 6 h. There were 4 downregulated and 3 upregulated carboxylesterase genes at 12 h, and 1 downregulated gene was upregulated at 6 h. Additionally, there were 7 downregulated genes and 1 upregulated gene at 24 h, and the upregulated genes were downregulated at 12 h. The differential expression of 60S ribosomal protein-encoding genes at different time points contrasted with that of cytochrome P450 genes (Figure 5a,b), with 4 and 23 DEGs at 6 h and 24 h, respectively, that were upregulated. The upregulated genes at 24 h were downregulated at 12 h. There were 2 genes that were upregulated, downregulated, and upregulated at 6 h, 12 h, and 24 h, respectively. There were two differentially expressed 60S ribosomal protein-encoding genes that were upregulated and downregulated at 6 h and 12 h, respectively. Among the remaining 120 60S ribosomal protein-encoding genes, there were 30 downregulated genes and 90 upregulated genes at 12 h.

#### 2.4.4. Expression of Genes Related to the Immune Defense Response

We screened genes related to “serpin”, “cecropin”, “chitinase”, and “heat shock protein”, which are involved in the immune defense response of *D. abietella* larvae after they feed on *Bt* 2913 (Figure 6d). We identified 2 cecropin-encoding genes that were downregulated only when the larvae had been fed *Bt* 2913 for 6 h. After feeding for 12 h and 24 h, 14 chitinase-encoding genes were identified, among which 8 upregulated genes and 4 downregulated genes were found at 12 h, 3 downregulated genes were found at 24 h, and 1 chitinase gene was differentially regulated at both the 12 h and 24 h. When larvae were fed *Bt* 2913 for 6 h, only 1 gene related to heat shock proteins was upregulated, which was downregulated at 12 h. After feeding on *Bt* 2913 for 12 h, *D. abietella* had 38 heat-shock-protein-encoding genes that were differentially expressed, including 12 downregulated genes and 26 upregulated genes. When the larvae were fed *Bt* 2913 for 24 h, only 4 downregulated and 1 upregulated heat-shock-protein-encoding genes were found, and 2 downregulated genes were upregulated, and 1 upregulated gene was downregulated at 12 h.

Among the genes associated with serpin inhibitors, 1 antichymotrypsin and 10 zonadhesin genes were downregulated at 6 h, among which the antichymotrypsin and 8 zonadhesin genes were still downregulated at 12 h. After feeding on *Bt* 2913 for 12 h, *D. abietella* had 8 upregulated and 2 downregulated antichymotrypsin-encoding genes, and only 2 genes encoding zonadhesins were upregulated, and 13 genes were downregulated at 12 h. In addition, 4 upregulated and 3 downregulated serine protease inhibitor genes and 3 NR-annotated “serpin” genes were identified at 12 h, and the downregulated serine protease inhibitors at 12 h were still downregulated at 24 h. A total of 3 antichymotrypsin genes were upregulated, 4 serine protease inhibitor genes were downregulated, and 1 serine protease inhibitor gene was upregulated after feeding on *Bt* 2913 for 24 h. 

### 2.5. Bioinformatic Analysis of Zonadhesin and Three Detoxification Enzymes in D. abietella

The deduced amino acid sequences of zonadhesin (PQ246119), GST (PQ260740), CYP450 (PQ260739), and CarE (PQ260738) were 346, 214, 372, and 583 amino acid residues, respectively. An analysis of the four amino acid sequences via SignalP4.1 and TMHMM-2.0 revealed that zonadhesin, GST, and cytochrome P450 lacked signal peptides and transmembrane segments. CarE lacks a signal peptide but has a transmembrane fragment at amino acid residues 21 to 43, suggesting that it may function at the cell membrane to bind and catalyze the hydrolysis of *Cry* and *Vip* toxins.

**Figure 7:**
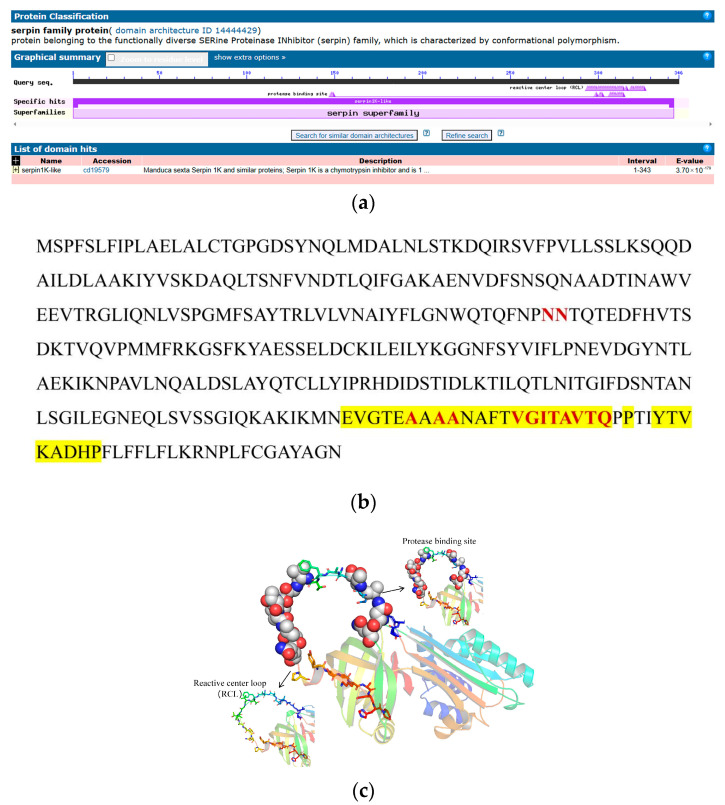
Bioinformatic analysis of zonadhesin in *D. abietella*. (**a**) Conserved zonadhesin structure domain; (**b**) sequence analysis of zonadhesin. The protease binding site is indicated in red. Yellow represents the reactive center loop (RCL); (**c**) protein tertiary structure of zonadhesin. The sphere representation area is a protease binding site. The stick representation area indicates the RCL.

NCBI Conserved Domain Search revealed a highly conserved serpin1K-like domain at amino acid residues 1 to 343, and the domain contains several motifs, such as a protease-binding site and a reactive center loop (RCL) (Figure 7a,b). Protein tertiary structure prediction was performed on zonadhesin, and the results showed that the structure of the model was reasonable (Figure S6). In the three-dimensional structure of zonadhesin, the area shown in the sphere representation is the protease-binding site, and the area shown in the stick representation is the RCL (Figure 7c). 

**Figure 8:**
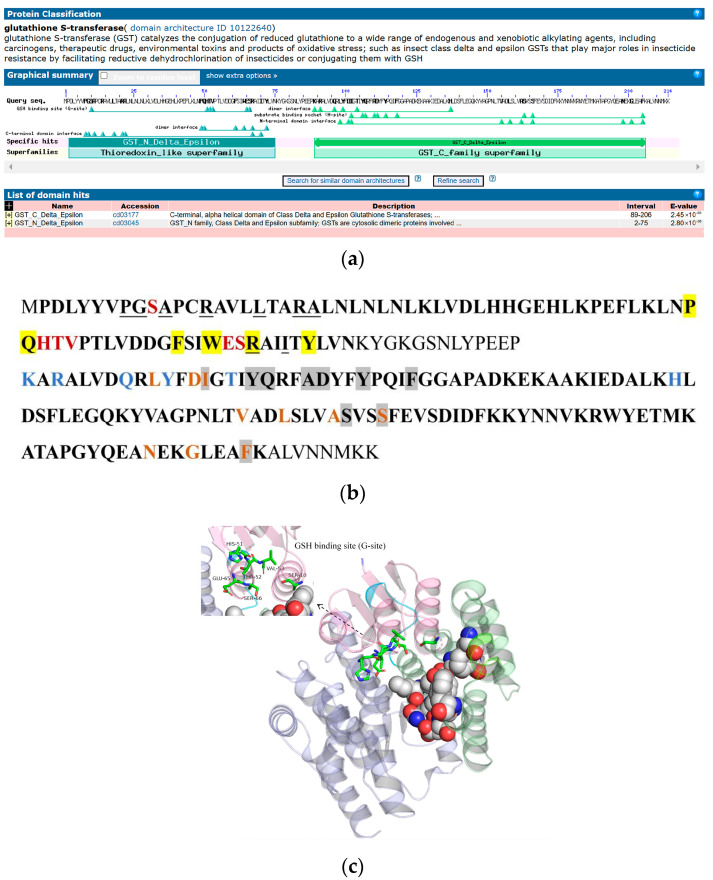
Bioinformatic analysis of GSTs in *D. abietella*. (**a**) Conserved GST structural domain; (**b**) sequence analysis of GSTs. Red represents the GSH-binding site (G-site). Yellow indicates the dimer interface. The underline indicates the C-terminal domain interface. GST-N-Delta-Epsilon is shown in bold. The dimer interface is shown in blue, the substrate-binding pocket (H site) is shown in gray, and the N-terminal domain interface is shown in yellow. The following part in bold indicates GST-C-Delta-Epsilon; (**c**) protein tertiary structure of GST. The pink color represents GST-N-Delta-Epsilon. GST-C-Delta-Epsilon is shown in green. The stick representation region is the G-site. The sphere representation area is the H-site.

GST contains two conserved domains, GST-N-Delta-Epsilon and GST-C-Delta-Epsilon, which are located at amino acid residues 2 to 75 and 89 to 206, respectively. The GST-N-Delta-Epsilon domain contains a GSH-binding site (G-site), a dimer interface, and a C-terminal domain interface. The GST-C-Delta-Epsilon domain contains a dimer interface, a substrate-binding pocket (H-site), and an N-terminal domain interface (Figure 8a,b). The protein tertiary structure of GST was predicted, and the results revealed that the predicted structure of the model was reasonable (Figure S7). In the three-dimensional structure of GST, the pink region corresponds to GST-N-Delta-Epsilon, and the stick representation region corresponds to the G-site. The green area is GST-C-Delta-Epsilon, and the sphere representation area is the H-site (Figure 8c). 

**Figure 9:**
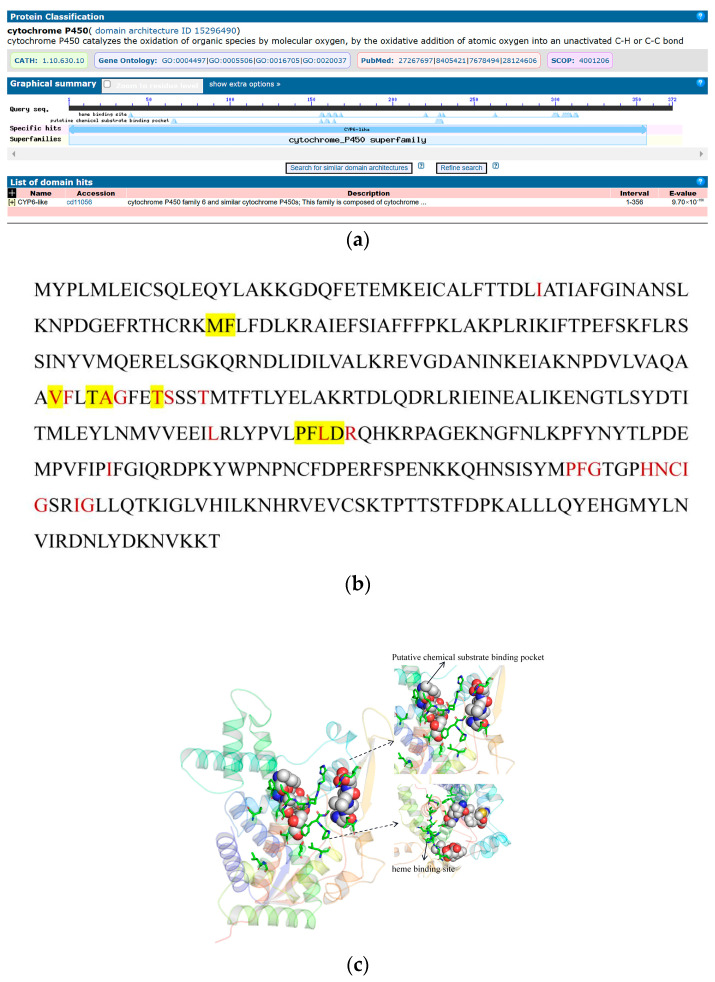
Bioinformatic analysis of CYP450 in *D. abietella*. (**a**) Conserved structure domain of CYP450; (**b**) sequence analysis of CYP450. Red represents a heme-binding site, and yellow represents a putative chemical substrate-binding pocket; (**c**) protein tertiary structure of CYP450. The stick representation region is a heme-binding site. The sphere representation area is a putative chemical substrate-binding pocket.

CYP450s at amino acid residues 1 to 356 contain a CYP6-like conserved domain structure. Some residues in the domain compose the heme-binding site and putative chemical substrate-binding pocket (Figure 9a,b). The protein tertiary structure of CYP450 was predicted, and the results revealed that the predicted structure of the model was reasonable (Figure S8). In the figure, the stick representation region in the 3D structure of CYP450 is a heme-binding site, and the sphere representation region is a putative chemical substrate-binding pocket (Figure 9c).

**Figure 10:**
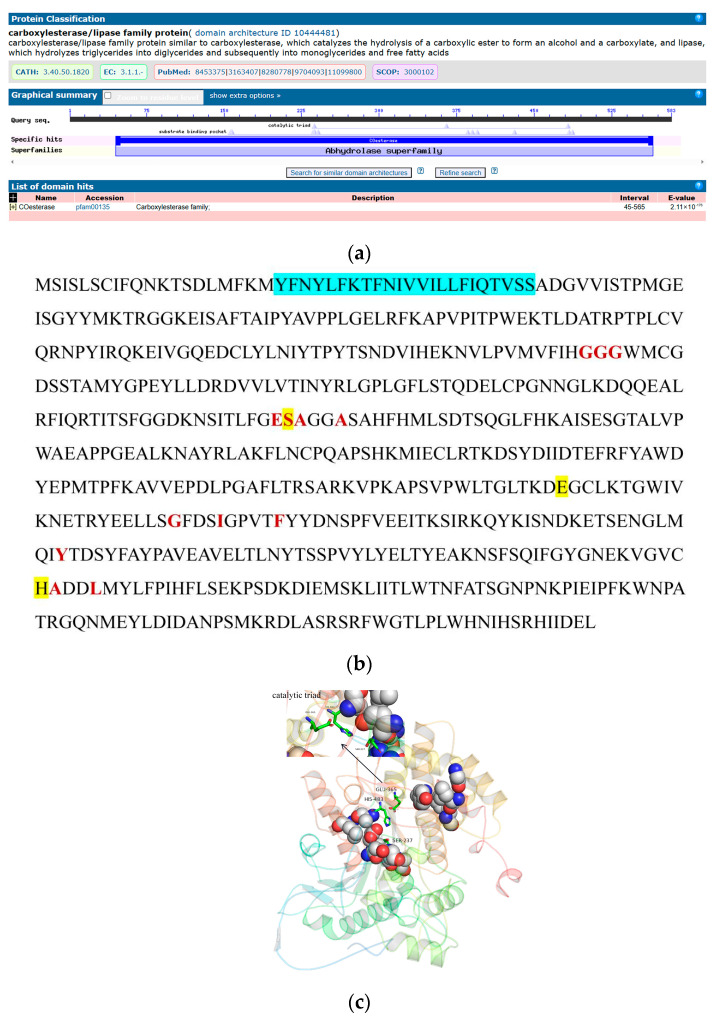
Bioinformatic analysis of CarE in *D. abietella*. (**a**) CarE conserved structural domain; (**b**) sequence analysis of CarE. Red indicates the substrate-binding pocket. Yellow indicates the catalytic triad. The transmembrane domain is shown in blue; (**c**) protein tertiary structure of CarE in. The stick representation region represents the catalytic triad. The sphere representation area represents the substrate-binding pocket.

Structural analysis of the protein sequences of the CarE domain revealed that amino acid residues 45 to 565 in the carboxylesterase family have a conserved COesterase domain structure, and the structural domain contains several motifs, such as a substrate-binding pocket and a catalytic triad. CarE has a transmembrane segment at amino acid residues 21 to 43 (Figure 10a,b). The protein tertiary structure of CarE was predicted, and the results revealed that the predicted structure of the model was reasonable (Figure S9). In the figure, the sticks labeled SER-237, GLU-365, and HIS-483 in the 3D structure of CarE compose the catalytic triad, and the sphere representation region is the substrate-binding pocket (Figure 10c).

### 2.6. Verification of Real-Time Fluorescence Quantitative PCR (qRT-PCR)

**Figure 11:**
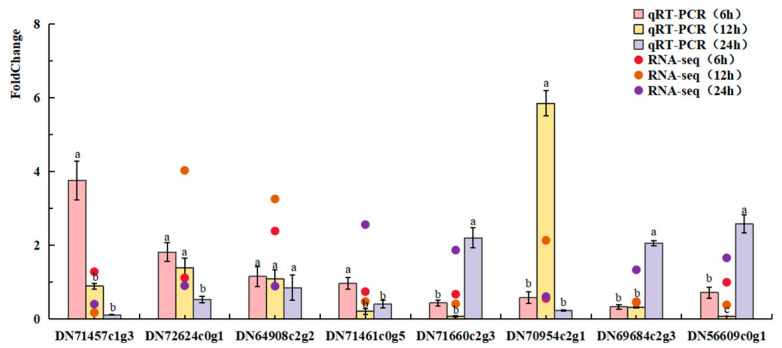
Quantitative real-time PCR (qRT-PCR) validation of selected DEGs from the midgut transcriptome of *D. abietella* larvae at different time points of infection with *Bt* 2913. Different lowercase letters of the same gene indicated that the difference between different time periods was statistically significant (Duncan test, *p* < 0.05).

To further verify the DEGs in the transcriptomic libraries derived from the midgut of *D. abietella* larvae at 6 h, 12 h, and 24 h after infection with *Bt* 2913, we selected eight DEGs, namely, DEGs annotated as “trypsin”, “GST”, “carboxylesterase”, “alkaline phosphatase”, and “cytochrome P450”, for quantitative analysis via qRT-PCR. RPS3 and EF-1-alpha, which are stably expressed in *D. abietella*, were selected as reference genes for data normalization. The results revealed that the expression levels of the selected DEGs were generally consistent with the transcriptome data. The changes in expression determined by mRNA sequencing were confirmed to be correct (Figure 11).

## 3. Discussion

### 3.1. Mechanism of Action of Bt 2913 on D. abietella Larvae

Serine protease (SP) and serine protease homolog (SPH) in insects are involved in digestion, development, immunity, and other processes [22]. These serine proteases contain a conserved catalytic triad of His, Asp, and Ser residues and are mainly divided into two classes, trypsin and chymotrypsin, depending on the restriction site [16,23]. Serine protease inhibitors are involved mainly in the immune response of insects, and mutually exclusive alternative splicing of the exons encoding the RCL can produce a variety of inhibitors with different inhibitory activities [24,25]. In our study, it was found that *Bt* 2913 carried the *Cry*1Ac, *Cry*2Ab, and *Vip*3Aa genotypes. *Vip*3A does not affect *Cry*1Ac or *Cry*2Ab2 binding, as shown by competitive binding assays [26]. We found that in the midgut of *D. abietella* larvae fed *Bt* 2913 for 6 h, trypsin levels tended to increase, chymotrypsin levels did not change, and serine protease inhibitor expression was downregulated. At 12 h of feeding, the serine protease expression tended to increase, chymotrypsin and antichymotrypsin expression was upregulated, and the expression of the other serine protease inhibitors tended to decrease. When the insects were fed for 24 h, the expression of chymotrypsin and other serine proteases tended to be downregulated, and the expression of serpin gradually stabilized. These results indicated that the period from 6 h to 12 h of *D. abietella* ingestion of *Bt* 2913 was the prototoxin activation stage. The corresponding *Cry* and *Vip* protoxins are activated mainly by trypsin and chymotrypsin. Studies have shown that after insects ingest *Bt*, trypsin and chymotrypsin in the insect midgut can further process the *Cry* prototoxin into the active toxin [27]. The *Vip*3Aa prototoxin can be activated by trypsin, but the degradation of the 62 kDa active core fragment of the *Vip*3A toxin is catalyzed mainly by cationic chymoproteinase-like peptidase [28]. This finding confirms our hypothesis: we speculate that when *D. abietella* ingests *Bt* 2913 for 12 h, although *Vip*3Aa is activated by trypsin, its 62 kDa toxin-active core fragment might be degraded by chymotrypsin.

Binding of the activated *Bt Cry* toxin to its specific receptors, which include alkaline phosphatase (ALP), aminopeptidase N (APN), ATP-binding cassette (ABC) transporters (ABCs), G-protein, and cadherin, is key to the virulence induced by the *Bt Cry* protein targeting insects [29,30,31,32]. In our study, the expression of the receptor proteins screened in the midgut transcriptome of *D. abietella* larvae at 6 h and 12 h of *Bt* 2913 infection was generally downregulated. Two ABCG genes, one APN gene, and one ALP-related gene were upregulated in the midgut transcriptome of the larvae after 12 h of *Bt* 2913 ingestion. The downregulation of cadherin expression was observed at 12 h and 24 h. However, the differential expression of genes related to NADH dehydrogenase occurred only at 12 h, and all were upregulated. In contrast, G-protein-coupled receptor (GPCR) and aminopeptidase-related genes were upregulated at 24 h. We speculate that in *D. abietella* larvae, beginning at 12 h after ingesting *Bt* 2913, the receptor protein begins to bind activated *Cry*/*Vip* toxins. Although some receptor-protein-related genes were downregulated to increase resistance to *Bt* 2913 after feeding on *Bt* 2913 for 12 h, some receptor proteins with high or low expression still have the potential to bind with the *Cry*/*Vip* toxin. Indeed, some studies have shown that the resistance of insects to *Bt* is related to the expression of receptor proteins; for example, compared with susceptible larvae, *Cry*1Ac-resistant pink bollworms present a 79- to 190-fold reduction in the transcription of a midgut cadherin gene [33]. In addition, cadherins in the larval midgut of Lepidoptera have been proposed to be important receptors for *Cry*1Ac, but cadherins in *H. armigera* have not been found to be functional receptors for *Cry*2Ab [32,34]. Therefore, the downregulation of cadherin expression in *D. abietella* may increase resistance to *Cry*1Ac, but whether it is a functional receptor for *Cry*2Ab needs to be further verified. According to the current understanding, α-amylase is not limited to its ability to aid insect digestion, so that they can absorb and utilize food in different environments [35]. α-Amylase has also been identified as a binding receptor for *Cry*4Ba, *Cry*11Aa, and *Cry*11Ba [36]. Therefore, we screened α-amylase as a potential receptor protein among the DEGs of the midgut transcriptome of *D. abietella* larvae after the ingestion of *Bt* 2913 and found that its expression was upregulated at both 6 h and 12 h; however, whether it is a receptor protein for the activated *Bt* 2913 toxin needs further analysis. Currently, the ABC transporters that act as *Bt* toxin receptors include mainly the A, B, C, D, and G subfamilies, such as ABCC2 and ABCG1 for *Cry*1Ab receptor proteins; ABCC2/3 and ABCG1 for *Cry*1Ac receptor proteins; and ABCA2 for *Cry*2Ab receptor proteins [37]. Studies on the *Cry*1Ac receptor protein in the midgut of *Plutella xylostella* (L.) larvae identified NADH dehydrogenase iron-sulfur protein 3 (NDUFS3) as a *Cry*1Ac-binding protein [38]. Therefore, we speculate that 2 ABCG-related proteins and 19 NADH dehydrogenases may bind to the *Cry*1Ac-activating toxin in *Bt* 2913. Although *Vip* toxins function in a similar way to *Cry* toxin, the receptor-protein-binding steps of *Vip*3Aa toxin and *Cry* toxin differ [39]. Reports have shown that *Vip*3A cannot bind with APN and cadherin-like proteins, but *Vip*3A does bind to different 80 and 100 kDa proteins in brush border membrane vesicles (BBMVs) [26,40,41,42]. Among these toxins, the *Vip*3Aa16 toxin can identify 55 and 100 kDa receptors in the BBMVs of *Spodoptera littoralis*. However, in the BBMVs of *Ephestia kuehniella*, it can bind to the 65 kDa receptors [40]. However, exploring the transcriptomic defense response of the *Agrotis ipsilon* to the *Vip*3Aa toxin revealed the differential expression of ALP, APN, ABC transporters, G-proteins, and cadherins. However, the receptor protein of *Vip*3Aa has not been further studied [43]. Therefore, whether the potential receptor proteins identified thus far are the receptor protein of *Vip*3Aa toxin still needs to be further explored.

### 3.2. Metabolic Detoxification and Immune Defense of D. abietella Larvae in Response to Bt 2913

The detoxification system of insects in response to exogenous substances can be divided into three stages. The first stage is biotransformation, which mainly involves the reduction in the activities of various exogenous substances by detoxification enzymes such as CYP450 and esterase. The second stage is metabolism, which is mainly performed by detoxification enzymes such as GSTs and UGTs to degrade the toxic byproducts of the first stage of metabolism. In the third stage, excretion, the bound toxin is exported to the extracellular space by transporters [44,45,46,47]. Therefore, CYP450, GST, esterase (e.g., CarE), and UGTs, which are important metabolic detoxification enzymes of insect resistance, often play important roles in the resistance to pathogenic microorganisms [19,48].

In the first phase, insect carboxylesterases participate in the detoxification of exogenous compounds by hydrolyzing carboxylester bonds [49]. When *Spodoptera exigua* (Hubner) were fed transgenic *Bacillus thuringiensis* (*Bt*) cotton for 1, 6, or 24 h, the CarE level in the moth was significantly lower than that in moths that did not consume *Bt*-impregnated cotton [50]. The third-instar larvae of *Ostrinia furnacalis* (Guenee) presented significantly lower carboxylate esterase activity after being fed transgenic *Bt* corn than the larvae fed control corn [51]. These results suggest that insect CarE may be involved in the detoxification of *Bt*. In our study, the number of DEGs related to CarE in the midgut of *D. abietella* after feeding on *Bt* 2913 gradually increased at 6 h, 12 h, and 24 h. In addition, the DEGs related to CarE were upregulated and downregulated in the larvae fed *Bt* 2913 for 6 h and 12 h, and most of the genes related to CarE were downregulated in the larvae fed *Bt* 2913 for 24 h. As indicated by biochemical analyses, resistant insect populations, whose esterase binds to both the *Bt* proprotein and the activated proprotein, prevent the toxin from binding to its receptor [2]. Therefore, we speculated that CarE in the midgut of *D. abietella* larvae may bind to the activated *Bt* 2913 toxin or participate in the degradation of the activated toxin. However, insect CYP450, as the main driver of exogenous substance metabolism and insect resistance, exerts its influence mainly via the insertion of an oxygen atom, which binds near the P450 heme iron center, thus catalyzing the oxidative modification of endogenous and exogenous substances [52]. In our study, we found that there were CYP450-related DEGs in the midgut of larvae after feeding on *Bt* 2913 for 6 h and 12 h, and the DEGs at 12 h were not only the most abundant but also both upregulated and downregulated. All the DEGs related to CYP450 were downregulated in the larvae after they were fed *Bt* 2913 for 24 h. Moreover, some studies have shown that the 60S ribosomal protein L18 is an important factor or carrier that promotes the accumulation of Rice stripe virus (RSV) in gray planthoppers [53]. A study of *Culex pipiens pallens* revealed that 60S ribosomal protein L22 (RPL22) in *C. pipiens pallens* could inhibit CYP450 6A1 activity [54]. In our study, the differential gene expression of the 60S ribosomal protein of *D. abietella* larva at different periods after feeding on *Bt* 2913 was essentially opposite to the changes in CYP450 expression, which corresponds to the above studies.

In the second stage, the UGT-mediated detoxification pathway is different from CYP450-mediated oxidative metabolic detoxification, which mainly catalyzes the binding of exogenous metabolites to UDP-glucose, thus producing more polar and water-soluble substrates for effective excretion [55,56,57]. The expression of 13 UGT-related genes as screened in the midgut of *D. abietella* larvae fed *Bt* 2913 for different durations. A total of 4 of the 5 UGT-related genes were upregulated in the midgut of the larvae fed *Bt* 2913 for 12 h. However, the DEGs related to UGTs were generally downregulated in the midgut of larvae fed *Bt* 2913 for 6 h and 24 h. We speculate that UGTs in the larval midgut are involved in the metabolic detoxification of *Bt* 2913. UGTs have been found in many insects and have been shown to detoxify a variety of pesticides. For example, *Diaphorina citri* (Hemiptera: Lividae) is a UGT involved in the detoxification of imidacloprid [58], and UGTs in *Chironomus kiiensis* have a metabolic effect on carbaryl, deltamethrin, and phoxim [59]. The gut UGTs of *Spodoptera frugiperda* larvae catalyze benzoxazinoid (BXD) detoxification reactions [60].

GSTs can catalyze the reduction in GSH to combine with exogenous substances after the first stage of biotransformation, thereby neutralizing the highly active nucleophilic sites of chemicals or increasing their water solubility and making them easy to excrete. GST may also directly affect the metabolism of toxic substances [61,62]. Its highly conserved amino terminal domain structure, which contains G-sites and amino acids, exerts important catalytic activity, and its carboxyl terminal domain structure and H-site interaction play important roles in substrate specificity [20,62]. In our study, GST-related genes were upregulated in the midgut of *D. abietella* after 6 h and 12 h of feeding on *Bt* 2913. The GST expression returned to normal levels at 24 h after *D. abietella* feeding on *Bt* 2913. These findings indicate that GSTs in the larval midgut directly metabolize *Bt* 2913 factors or participate in the second stage of the detoxification system, when *Bt* 2913 has been ingested for 6 h. Currently, the detoxification effect of GST on insecticides has been extensively studied, and many GSTs have been shown to interact with and metabolize insecticides [63].

The final stage mainly involves ABCs, and other transport proteins are involved in the first phase and/or the second stage after metabolic toxins are exported by the cells; however, the metabolites of the first and second stages can also be directly eliminated without modification [20]. ABCs are transmembrane proteins that are involved in insecticide metabolic detoxification and can combine with *Bt*-activated toxins to induce perforation [37]. In our study, 32 genes related to ABCs were found to be differentially expressed in the midgut of *D. abietella* larvae after they were fed *Bt* 2913 for 12 h, and only 2 genes related to ABCG were upregulated. We speculate that the midgut of larvae enhances resistance to *Bt* 2913 by downregulating ABC-related genes and that upregulated ABCGs act as receptor proteins for the *Bt* 2913 *Cry*1Ac-activating toxin. The ABCG transcripts in the midgut of Asian corn borers (*Ostrinia furnacalis*) resistant to *Cry*1Ab and *Cry*1Ac are depleted compared with those in susceptible *O. furnacalis*; however, the ABCG-related genes encode the receptor proteins of *Cry*1Ab- and *Cry*1Ac-activated toxins [37,64]. This finding is consistent with our inference. Although ABCs are the receptor proteins of *Bt* toxins: when the ABC transporter is inactivated, it can reduce binding to *Bt*-activated toxins, thereby increasing the resistance of target insects to *Bt* [65,66]. For example, a silkworm, *Bombyx mori*, is resistant to the *Bt* toxin *Cry*1Ab when an amino-acid-altering mutation occurs in the ABC transporter gene [67]. Combined mutations of ABCC2 and ABCC3 in *Plutella xylostella* can increase resistance to *Cry*1Ac [68]. Therefore, ABCs can moderately affect the resistance of *D. abietella* larvae to *Bt* 2913.

Hsp90 can protect the *Cry*1A proprotein from degradation and enhance binding to the receptor, thereby increasing *Bt* toxicity [69]. Our study of the midgut transcriptome of *D. abietella* 12 h after feeding on *Bt* revealed 12 downregulated and 26 upregulated heat-shock-protein-related genes. We speculated that Hsps in the larval midgut could increase *Bt* 2913 toxicity and protect the *Cry*1Ac proprotein from degradation. A study of the *Aedes aegypti* larval midgut revealed that when RNAi was used to silence the expression of Hsp90, larvae were resistant to *Cry*11Aa [70]. These findings also indicate that Hsps can increase *Bt* virulence.

On the basis of the above findings, we speculated that the metabolic detoxification and immune defense response of *D. abietella* larvae to *Bt* 2913 mainly began at 12 h after the larvae were fed *Bt* 2913 and could mediate the resistance to the activated *Bt* 2913 toxin. However, owing to the expression of 60S ribosomal protein and Hsp-related genes, the resistance of the larvae to *Bt* 2913 was weakened. At 24 h, the larvae were less resistant to *Bt* 2913.

## 4. Materials and Methods

### 4.1. Identification of the Genotype of Bacillus thuringiensis 2913 and Its Crystal Metabolism

*Bacillus thuringiensis* 2913 (*Bt* 2913) was purchased from the China Forestry Culture Collection Center. The strains were inoculated in LB solid culture for intensive activation and then transferred to 50 mL of LB liquid medium after 24 h. The LB liquid medium was composed of tryptone (10 g/L), yeast extract (5 g/L), and NaCl (10 g/L). To prepare 1 L of LB solid medium, which maintains the same formulation as the liquid medium, an additional 15–20 g of agar powder is required. The bacterial mixture was cultured for 24 h, 26 h, 30 h, and 48 h in a constant-temperature shaker at 30 °C and 200 r/min, and 0.5% alkaline compound red solution was used for staining microscopy to observe the metabolic activity of parasporal crystals. The remaining bacterial culture was used for the *Cry* genotype identification experiments. Afterward, the *Cry* genotype of *Bt* 2913 was identified via PCR-RFLP according to the system used by Kuo and Seifinejad et al. [71,72]. Primer sequences were synthesized by Sangon Biotech (Shanghai) Co., Ltd. (Shanghai, China). One milliliter of bacterial culture was centrifuged at 12,000 r/min for 2 min. After the supernatant was discarded, the bacteria were ground in liquid nitrogen. After grinding, genomic DNA was extracted according to the instructions of the TIANamp Bacteria DNA Kit (Tiangen Biotech (Beijing) Co., Ltd., Beijing, China). Genomic DNA was used as a template for PCR amplification. The total amplification system volume was 25 μL: 12.5 μL of 2× Rapid Taq Master Mix (Vazyme Biotech Co., Ltd., Nanjing, China), 8.5 μL of ddH_2_O, 1 μL of each primer, and 2 μL of DNA. The amplification procedure was as follows: predenaturation at 94 °C for 3 min; 30 cycles of denaturation at 94 °C for 30 s; annealing at 55 °C for 30 s; extension at 72 °C for 2 min; and extension at 72 °C for 10 min. The PCR amplification products were detected via agarose gel electrophoresis at a 1% concentration, and the PCR amplification products were digested with a 30 μL digestion system: 10 μL of PCR product, 1 μL of restriction enzyme (Thermo Fisher Scientific, Waltham, MA, USA), 3 μL of 10× Tango buffer, and 15 μL of ddH_2_O. After 2.5 h of cleavage in a PCR instrument at 37 °C, the digestion results were checked via 1% agarose gel electrophoresis. The PCR products were subsequently sent to Heilongjiang Genesoul Technology Co., Ltd., Harbin, China for bidirectional sequencing, and the sequencing results were compared via NCBI BLAST (https://blast.ncbi.nlm.nih.gov/Blast.cgi) (accessed on 31 October 2023).

### 4.2. Experimental Insect Rearing and Sample Preparation

The fifth instar larvae of *D. abietella* larvae were collected from Hailin, Heilongjiang Province. The larvae were starved for 2 days in an incubator at a temperature of 25 ± 1 °C before infection. *Bt* 2913 was cultured at 30 °C and 200 r/min on a constant-temperature shaker for 30 h, after which 0.5% Tween 80 solution was added to dilute the bacterial mixture to 1 × 10^9^ CFU/mL for infection experiments. First, the artificial feed was soaked in diluted *Bt* 2913 solution for 30 s and then dried. The dried artificial feed and 100 healthy larvae of similar weight and appearance were subsequently placed in a clear 24-well plate. The larvae in the control group were fed a diet soaked in sterile LB liquid medium with the same volume of Tween 80 solution as in the treatment group, and the other manipulations of the control group were the same as those described above. The test was repeated 3 times. Then, larvae in the treatment group and the control group were collected at 3 different time points of feeding (6 h, 12 h and 24 h), and the collected larvae were dissected on a clean bench. The dissected larval midguts were allocated into 1.5 mL sterile, enzyme-free centrifuge tubes, with each tube containing 30 midguts. These samples were subsequently flash-frozen in liquid nitrogen and stored at −80 °C for subsequent transcriptome sequencing and real-time fluorescence quantitative PCR analysis.

### 4.3. RNA Extraction, Library Construction, and Sequencing

In accordance with the instructions of the Total RNA Extraction Kit (TRIzol), total RNA was extracted from larval midgut tissues treated with *Bt* 2913 for 6 h, 12 h, or 24 h and from the corresponding control groups. The concentrations of the RNA solutions obtained were determined using a Qubit 2.0 RNA test kit on an Invitrogen Qubit 2.0 fluorometer. RNA integrity and genome contamination were tested via 1% agarose gel electrophoresis. The total RNA from the *D. abietella* midgut tissue samples was subsequently subjected to mRNA purification and fragmentation, double-stranded cDNA synthesis, cDNA fragmentation, magnetic bead purification, fragment sorting, and library amplification via the 3′−end polyA structure of messenger RNA and related molecular biology techniques. After detection and quality control, a sequencing library suitable for the Illumina platform was finally obtained. An Illumina NovaSeq 6000 was used for sequencing by Sangon Biotech (Shanghai) Co., Ltd. The quality of the original sequencing data was evaluated via FastQC (version 0.11.2), and quality trimming was performed via Trimmomatic (version 0.36) [73] to obtain relatively accurate and useable clean data.

### 4.4. De Novo Assembly and Sequence Annotation

The clean reads were assembled de novo into transcripts via Trinity (version 2.4.0) [74], with the parameter min_kmer_cov 2 and the remaining parameters set to default values. Trinity’s workflow is divided into three primary steps: first, Inchworm is used to assemble the RNA-seq read data into unique sequences; second, Chrysalis is used to cluster contigs generated in the previous step; then, Bruijn graphs are constructed for each class; finally, these Bruijn graphs are processed with Butterfly, and paths are found according to reads and paired reads in the graphs, thus yielding full-length transcripts with variable splicing and separation of paralogs. Redundancy was removed from the transcripts obtained by the Trinity assembly, and the longest transcript in each transcript cluster was selected as the unigene to serve as the reference sequence for subsequent analysis. Bowtie2 (version 2.3.2) [75] was used to compare the read sequence after quality control with the reference sequence, the mapping information was processed, and the comparison results were counted via RSeQC (version 2.6.1) [76].

For sequence annotation, NCBI Blast+ (version 2.60) [77] was used to compare unigenes with the CDD (Conserved Domain Database), KOG (Clusters of Orthologous Groups for EuKaryotic Ortholog Groups), NR (NCBI nonredundant protein sequences), NT (NCBI nucleotide sequences), PFAM (Protein Family), and GO (Gene Ontology) databases to obtain functional annotation information. Moreover, by comparing unigenes with the NR database, it was possible to view the similarity of the transcript sequence of a species to that of a similar species, as well as the functional information of homologous sequences. The KEGG (Kyoto Encyclopedia of Genes and Genomes) annotation information of the transcripts was obtained via KAAS (version 2.1) [78], and CDS prediction of the unigenes was performed via TransDecoder (version 3.0.1).

### 4.5. Differential Expression of Genes in the Midgut Tissues of Bt 2913-Treated D. abietella Larvae at Different Time Points

To explore the mechanism of the *D. abietella* larva response to *B. thuringiensis*, we used Illumina NovaSeq 6000 sequencing to identify upregulated or downregulated differentially expressed genes (DEGs) in the midgut tissues of larvae infected with *Bt* 2913 for 6 h, 12 h, or 24 h. In RNA-seq analysis, gene expression levels can be estimated by counting read sequences that localize to genomic regions or gene exon regions. Salmon (version 0.8.2) [79] was used to calculate gene expression, and the transcripts per million (TPM) value was calculated. We used DESeq2 (version 1.12.4) [80] from an R language package for differential analysis and obtained significantly differentially expressed genes between the 6 h, 12 h, and 24 h *Bt* 2913-treated groups and the corresponding control group. The screening criteria were set as a q value < 0.05 and |fold change| > 2. The q value is the p value after correction. Then, clusterProfiler [81] (version 3.0.5) and topGO (version 2.24.0) (https://bioconductor.org/packages/release/bioc/html/topGO.html, accessed on 9 October 2024, DOI:10.18129/B9.bioc.topGO) in the R package were used for enrichment analysis of KEGG pathways and GO terms, respectively, to further identify the most relevant biological pathways among the groups.

### 4.6. Screening of Genes Associated with the Response of D. abietella Larvae to Bt 2913 Infection in Midgut Tissue

To identify the genes associated with the response of *D. abietella* larvae to *Bt* 2913 infection, pairs of *D. abietella* larvae infected for 6 h, 12 h, or 24 h were compared with the corresponding controls. On the basis of the infection mechanism of *B. thuringiensis* and the reported genes involved in *Cry* toxin activation, potential *Bt* toxin receptor-protein binding, metabolic detoxification, and immune defense, we conducted gene screening on the obtained DEG data at different time points. The screening method was mainly based on NR annotations of the DEG data and was further determined on the basis of combined CDD, PFAM, and KOG annotations. These terms include “trypsin”, “chymotrypsin”, “serine protease”, “cadherin”, “ATP-binding cassette transporter (ABC transporter)”, “aminopeptidase”, “alkaline phosphatase”, “G-protein coupled receptor”, “alpha-amylase”, “antichymotrypsin”, “zonadhesin”, “serine protease inhibitor”, “UDP-glucuronosyltransferase”, “glutathione S-transferase”, “cytochrome P450”, “carboxylesterase”, “NADH dehydrogenase”, etc. All the screened candidate genes needed to be manually confirmed with NCBI BLASTx to facilitate the subsequent focus on the candidate DEGs at the three time points. These candidate genes may be involved in the overall response to *Bt* 2913 infection in the *D. abietella* larva midgut. TBtools-II v1.113 software was used to construct a heatmap [82].

### 4.7. Bioinformatic Analysis of Zonadhesin and Three Detoxification Enzymes

The amino acid sequences of Zonadhesin and three detoxification enzymes (GST, CYP450 and CarE) were identified via ORFfinder (https://www.ncbi.nlm.nih.gov/orffinder/) (accessed on 5 February 2024). The obtained amino acid sequences were confirmed via BLASTp alignment against the UniProtKB/SwissProt database of NCBI (https://blast.ncbi.nlm.nih.gov/Blast.cgi) (accessed on 5 February 2024). The signal peptides and transmembrane domains were analyzed via SignalP 4.1 (https://services.healthtech.dtu.dk/services/SignalP-4.1/) (accessed on 5 February 2024) and TMHMM-2.0 (https://services.healthtech.dtu.dk/services/TMHMM-2.0/) (accessed on 5 February 2024), respectively. In addition, the detected protein sequences were subjected to domain analysis via NCBI Conserved Domain Search (https://www.ncbi.nlm.nih.gov/Structure/cdd/wrpsb.cgi) (accessed on 5 February 2024). The protein tertiary structures of zonadhesin and three detoxification enzymes were obtained using templates of cytochrome P450 from *Musca domestica* (T1PFB2.1.A, 71.74% identity), carboxylic ester hydrolase from *Helicoverpa armigera* (D5G3G2.1.A, 71.65% identity), glutathione S-transferase delta of the silk moth (3vk9.1.A, 85.85% identity), and serpin K of *Manduca sexta* (1sek.1.A, 41.25% identity), respectively, employing online SWISS-MODEL software (https://swissmodel.expasy.org/) (accessed on 6 February 2024). The rationality of the 3D structure model was evaluated via SAVES v6.0 (https://saves.mbi.ucla.edu/) (accessed on 6 February 2024). The resulting PDB files were visualized via the PyMOL Molecular Graphics System [83].

### 4.8. Validation by Real-Time Fluorescence Quantitative PCR (qRT-PCR)

Total RNA was extracted from larval midgut tissues of *Bt* 2913-infected and uninfected larvae at 6 h, 12 h, and 24 h, according to the instructions of the EASYspin Plus Tissue/Cell RNA Rapid Extraction Kit (Aidlab Biotechnologies, Ltd., Beijing, China). The concentration and purity of the resulting RNA solution were determined via a Nano-300 microspectrophotometer (Hangzhou Allsheng Instruments Co., Ltd., Hangzhou, China), and the integrity of the RNA and degree of genomic contamination were determined via 1% agarose gel electrophoresis. The obtained RNA was stored at −80 °C for later use. cDNA was obtained via reverse transcription from 1 μg of total RNA according to the instructions of the PrimeScript™ IV 1st Strand cDNA Synthesis Mix (Takara Biomedical Technology (Beijing) Co., Ltd., Beijing, China), and the obtained cDNA was stored at −20 °C for subsequent use. Gene-specific primers were designed according to the sequence template of the 8 differentially expressed genes via Primer Premier 5.0 software. The primers used were synthesized by Sangon Biotech (Shanghai) Co., Ltd. (Table S7). Real-time fluorescent quantitative PCR (qRT-PCR) was performed using KOD SYBR^®^ qPCR Mix (TOYOBO (SHANGHAI) BIOTECH Co., Ltd. (Shanghai, China)) on a CFX96 fluorescence quantitative PCR instrument (Bio-Rad, Hercules, CA, USA). The qRT-PCR results were analyzed via Bio-Rad CFX Maestro software V2.2 to determine the eligibility of each primer pair. cDNA amplification was performed via qRT-PCR in a 20 μL mixture containing 1 μL of cDNA, 1 μL of each (forward and reverse) primer (10 μM), 10 μL of KOD SYBR^®^ qPCR Mix, and 7 μL of ddH_2_O. EF-1-alpha and ribosomal protein S3 (RPS3) [12] were used as reference genes. The qRT-PCR amplification program was as follows: predenaturation at 98 °C for 2 min; 40 cycles of denaturation at 98 °C for 10 s, annealing at 60 °C for 10 s, and extension at 68 °C for 30 s; 65 °C for 1 min; and 95 °C for 15 s for plate reading. All the experiments were independently conducted three times. The 2^−ΔΔCT^ method was used to calculate the relative mRNA expression in the *D. abietella* midgut. IBM SPSS Statistics 26 was used for one-way ANOVA analysis of variance (Duncan’s test, *p* < 0.05).

## 5. Conclusions

On the basis of the above discussion, we conclude that feeding on *Bt* 2913 from 6 h to 12 h is the stage of the corresponding *Cry* and *Vip* protoxins are activated mainly by trypsin and other serine proteases in *D. abietella*. When *Bt* 2913 was ingested for 12 h, the increase in antichymotrypsin expression led to a decrease in chymotrypsin expression. However, many CarE and CYP450 genes in the midgut were involved in the metabolic detoxification of *Bt* 2913 at the first stage. However, the 60S ribosomal protein has inhibitory effects on CYP450, affecting the metabolic detoxification of *D. abietella* larvae in the midgut. Unlike other detoxification enzymes, the expression of GSTs was upregulated from 6 h to 12 h after feeding on *Bt* 2913. Additionally, GSTs in the midgut of *D. abietella* larvae are involved in the second stage of metabolic detoxification and direct metabolic detoxification. They begin to metabolize toxic substances by catalytically reducing glutathione (GSH) from 6 h to 12 h after being fed *Bt* 2913. UGTs catalyze the combination of the toxic byproducts of the first stage and UDP-glucose for metabolic detoxification when the larvae are fed *Bt* 2913 for 12 h. Interestingly, however, ABCs in the midgut of *D. abietella* larvae do not play a role in removing metabolized toxins from the cell; instead, they enhance resistance to *Bt* 2913 by downregulating genes related to ABCs. Moreover, the resistance of the *D. abietella* larvae to *Bt* 2913 further increased via the downregulation of ABCs and other receptor proteins. In addition, the massive upregulation of Hsp-related genes also protected the *Cry*1A protoprotein from degradation within *Bt* 2913. Therefore, when the larvae were fed *Bt* 2913 for 12 h, receptor proteins such as ALP and APN in the midgut began to bind to the activated *Bt* 2913 toxin. A total of 2 ABCG-related genes and 19 NADH dehydrogenases may bind to the *Cry*1Ac-activating toxin in *Bt* 2913. Cadherin binds to *Cry*1Ac in *Bt* 2913, but whether it is a functional receptor for *Cry*2Ab and *Vip*3Aa needs to be further verified. However, at 24 h, according to the changes of genes related to detoxification metabolism, the resistance of *D. abietella* larvae to *Bt* 2913 was weakened. At 24 h, we speculate that there is already a large amount of activated toxin in the larval midgut that binds to receptor proteins, such as aminopeptidase, in the midgut to form perforating complexes, resulting in the death of *D. abietella*.

## Data Availability

All data mentioned in this paper are available at the National Center for Biotechnology Information (NCBI) with the BioProject no. PRJNA1151836.

## Supplementary Materials

The following supporting information can be downloaded at: https://www.mdpi.com/article/10.3390/ijms252010921/s1.
