# Supplementary material for: Transcriptomic Analysis of the Response of the Dioryctria abietella Larva Midgut to Bacillus thuringiensis 2913 Infection
Source: Int J Mol Sci. 2024 Oct 10;25(20):10921. doi: 10.3390/ijms252010921 (PMC11507524; doi:10.3390/ijms252010921)
Supplement: Supplementary file 1 [file ijms-25-10921-s001.zip › Back matter.pdf]

Supplementary Materials

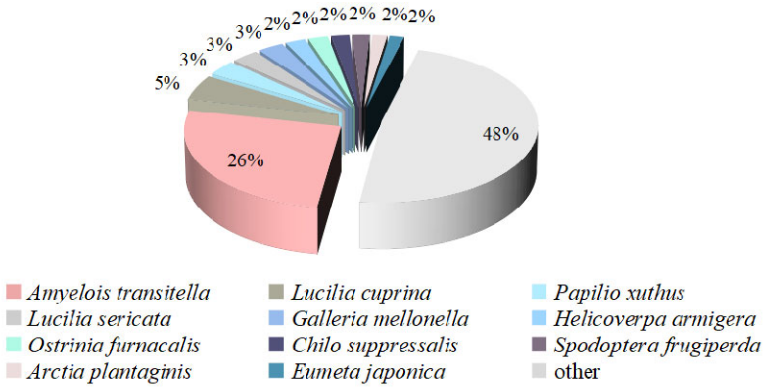

Figure S1. NR database homologous species classification.

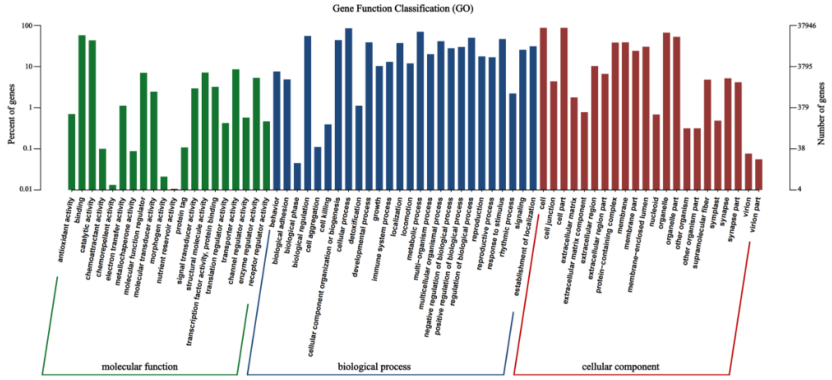

Figure S2. Gene function classification (GO).

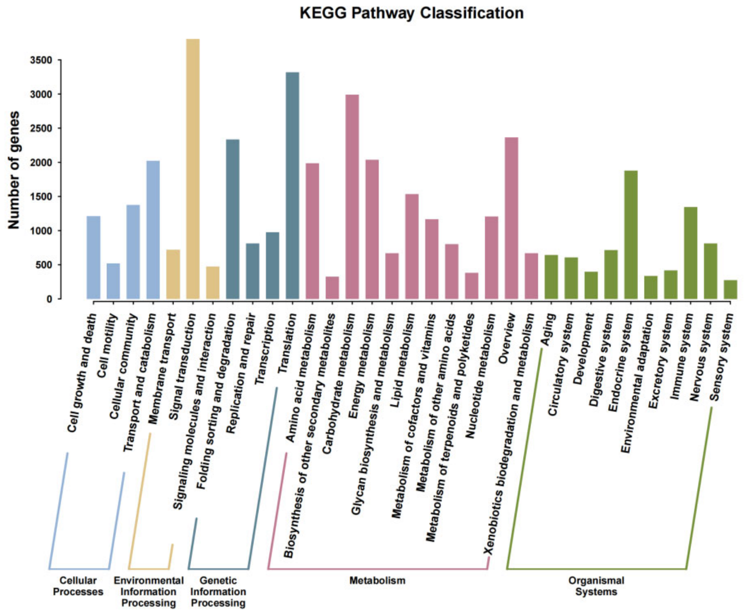

Figure S3. KEGG pathway classification.

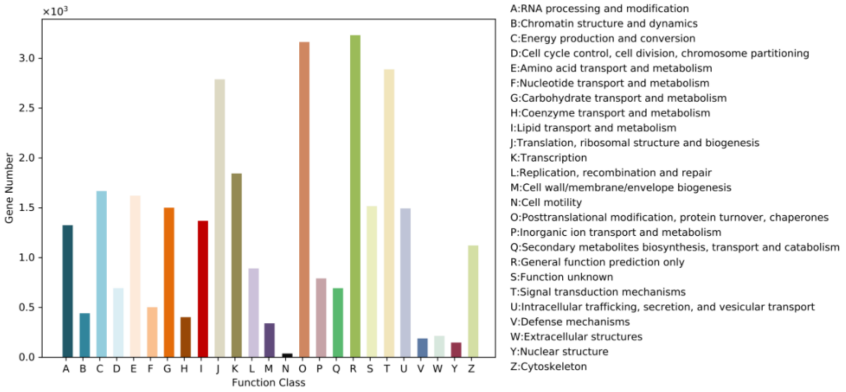

Figure S4. KOG function classification.

(a)

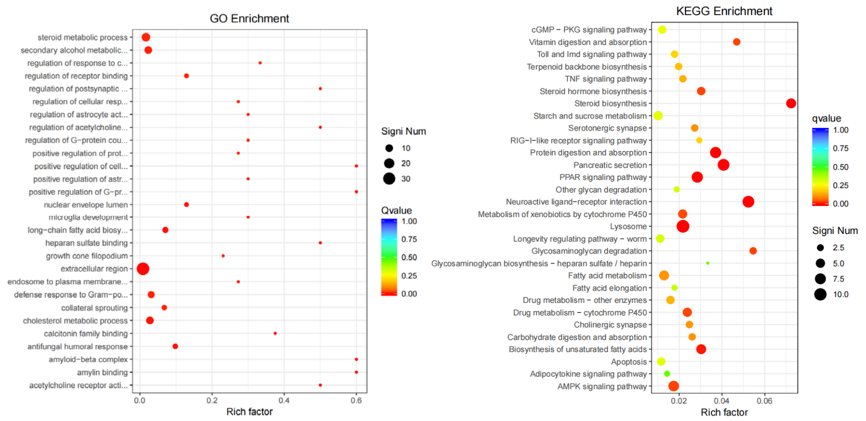

(b)

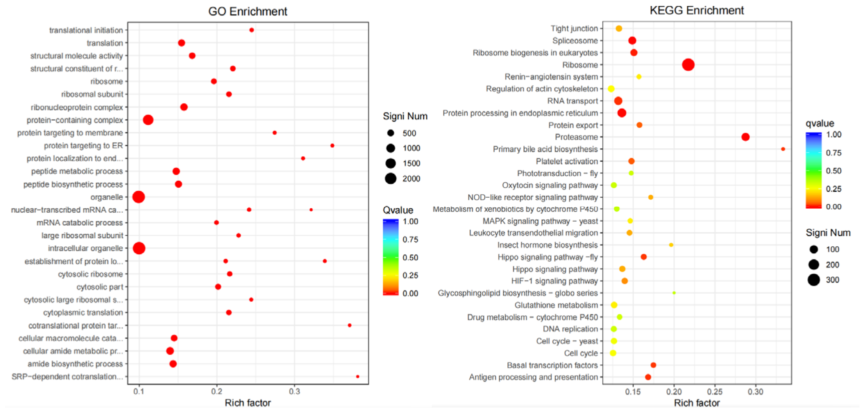

(c)

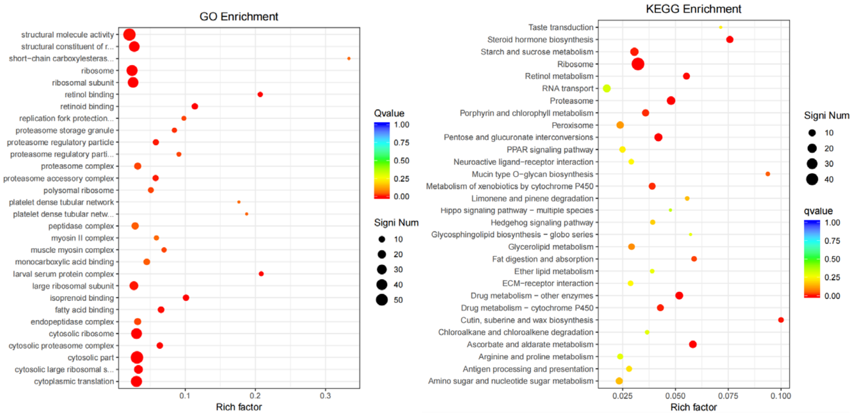

Figure S5. Scatter plots of GO and KEGG enrichment at different time periods in the larvae of *D. abietella* larvae infected with *Bt* 2913. (a), (b), and (c) show the GO and KEGG enrichment in the midgut of the *D. abietella* larvae fed on *Bt* 2913 for 6 h, 12 h, and 24 h, respectively.

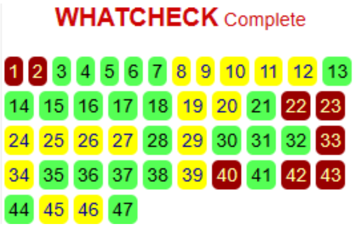

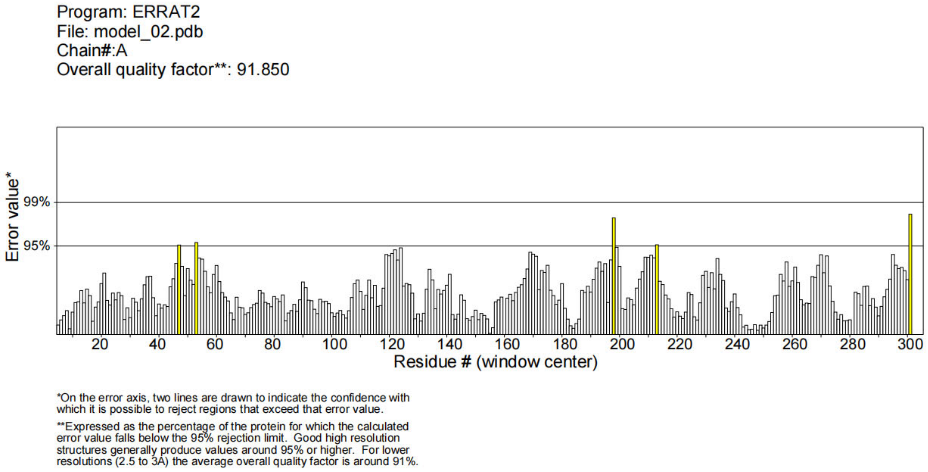

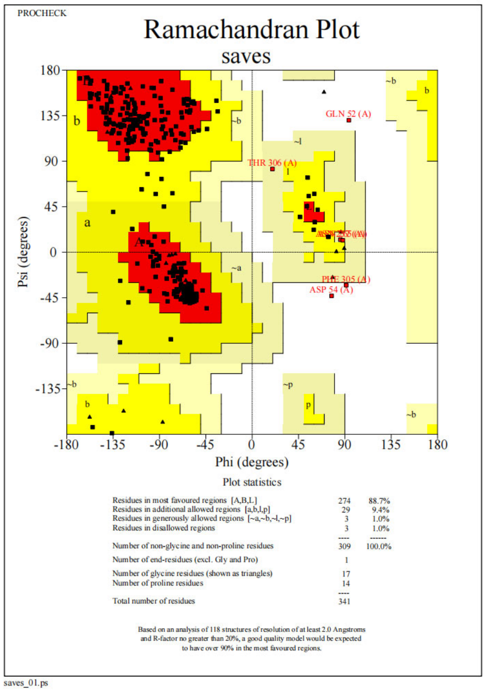

Figure S6. Protein tertiary structure reasonable prediction was performed on zonadhesin.

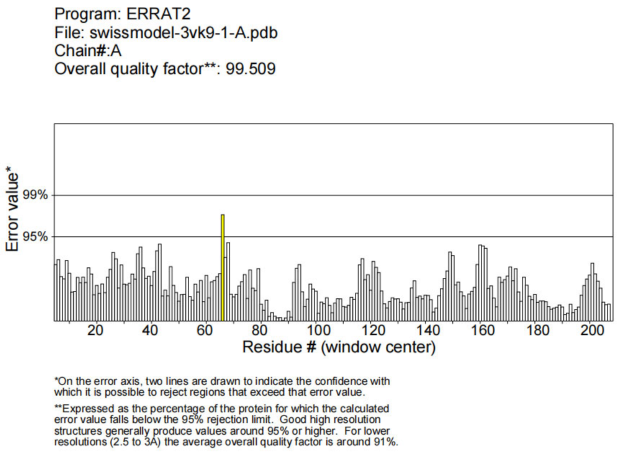

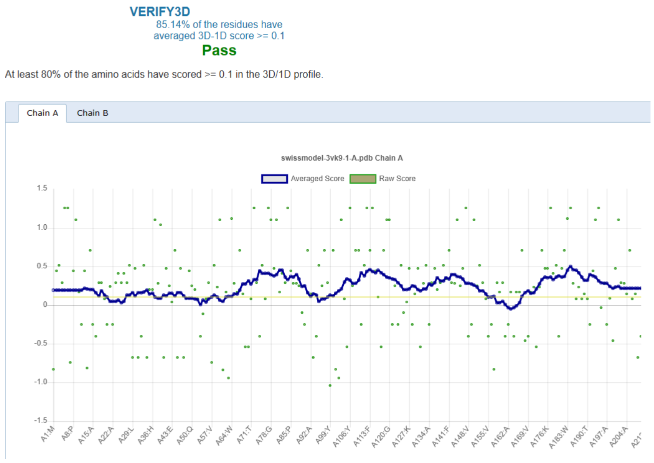

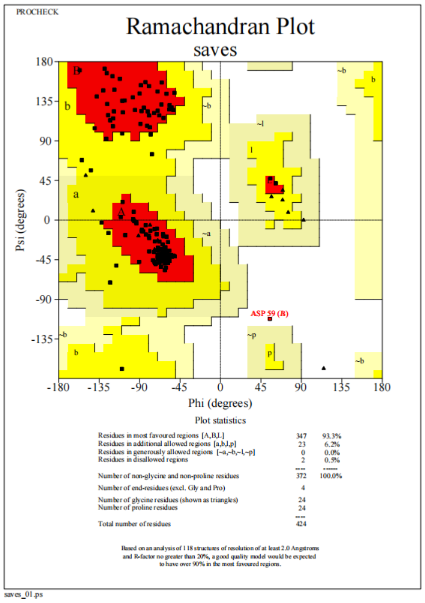

Figure S7. Protein tertiary structure reasonable prediction was performed on GST.

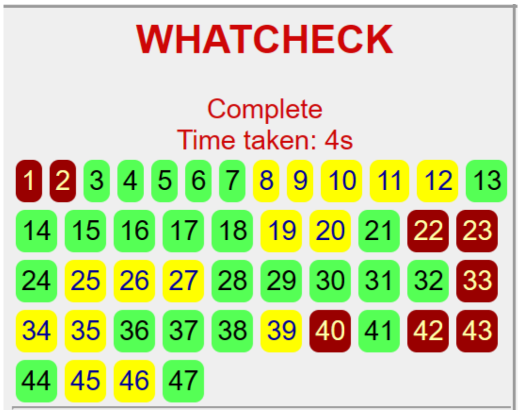

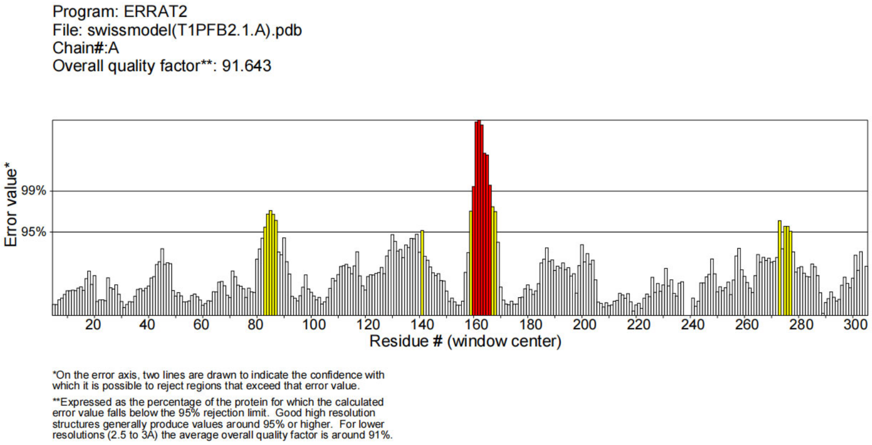

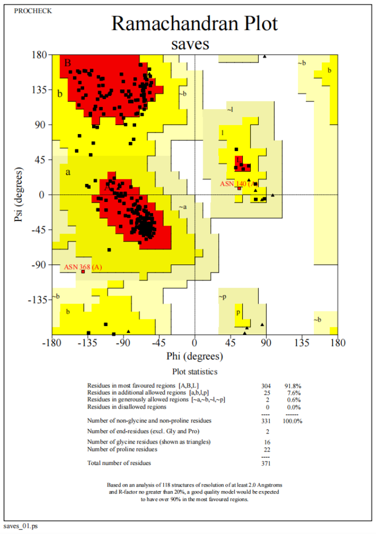

Figure S8. Protein tertiary structure reasonable prediction was performed on CYP450.

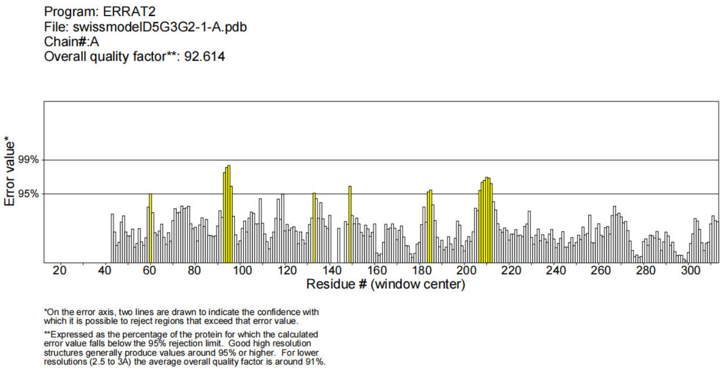

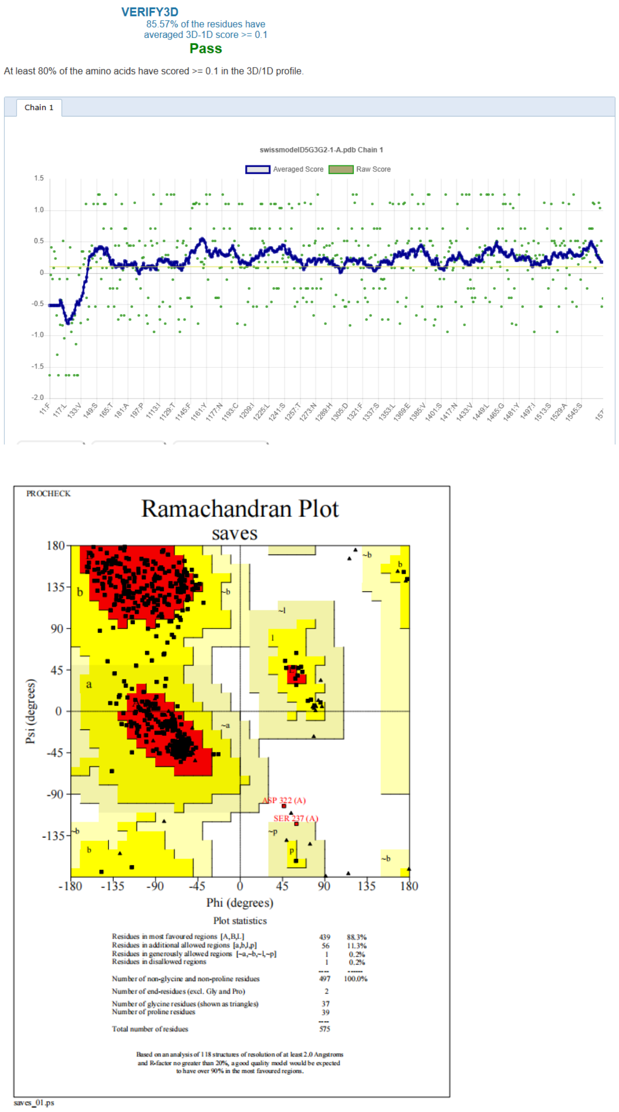

Figure S9. Protein tertiary structure reasonable prediction was performed on CarE.

Table S1. *Bt Cry* universal primer and *Vip*3Aa specific primer.

Gene	Primers	Sequences（5′-3′）
cry1	K5un2	AGGACCAGGATTTACAGGAGG
	K3un2	GCTGTGACACGAAGGATATAGCCAC
	K3un3	CCTCCTGTAAATCCTGGTCCT
	K5un3	CAATGCGTACCTTACAATTGTTTAAGTAAT
cry2	S5un2	GGAAGAACTACTATTTGTGATGC
	S3un2	AATAGTTTGAATTACCGCGAGC
cry3	S5un3	CGAACAATCGAAGTGAACATGATAC
	S3un3	CATCTGTTGTTTCTGGAGGCAAT
cry4	S5un4	GTGTCAAGAGAACCAACAGTATG
	S3un4	ACTAAGTCTCCTCCTGTATGACCAG

cry5	S5un5	ATTGGAGGTGGTATTGCTGATAC
	S3un5	ATAAGATGAAGACAGTGCTGGTGGTGG
cry6	S5un6	CAACAAATCCTAGCAATGGTC
	S3un6	TAGAGAGTGGAACGACTTTACC
cry7	S5un7	GGATATGAAGATAGTAATAGAAC
	S3un7	GCTGTAGCATGACATAATCGATG
cry8	S5un8	CGGCAAACTTAGTAGAATGC
	S3un8	CTGACTGATTTCCACCATCACG
cry9	S5un9	AGGACCAGGATTTACAGGAGG
	S3un9	CCCAATGCGAAAGAACTAAG
vip3Aa	SPvip3A(+)	CCTCTATGTTGAGTGATGTA
	SPvip3A(-)	CTATACTCCGCTTCACTTGA

Table S2. PCR amplification products and restriction length polymorphisms.

**Genes**	**PCR amplification products（K5un2/K3un2)（bp）**	**Digested with PstI and XbaI**
cry1Aa	1635	1117,518
cry1Ab	1557	1039,518
cry1Ac	1641	322,801,518
cry1Ad	1635	723,518,238,140,16
cry1Ae	1635	961,518,140,16
cry1Af	待定	1033,待定
cry1Ah	1581	741,518,322
cry1Ai	1641	661,518,322,140
cry1Ba	1686	1015,655,16
cry1Bb	1683	624,555,238,150,16
cry1Bc	1683	1449,238
cry1Be	1685	1014,655,16
cry1Ca	1671	758,423,239,140,95,16
cry1Cb	1632	958,518,140,16
cry1Da	1620	962,518,140

cry1Db	1620	946,518,140,16
cry1Ea	1635	743,518,218,140,16
cry1Eb	1632	974,658
cry1Fa	1635	961,518,140,16
cry1Fb	1632	732,423,238,140,94
cry1H	1626	802,518,150,140,16
cry1Hb	1620	946,658,16
cry1Ja	1629	777,515,238,99
cry1Jb	1622	729,655,166,81
cry1K	1632	888,513,156,81,73

**Genes**	**PCR amplification products（S5un2/S3un2)（bp）**	**Digested with HincII and MspI**
cry2Aa	1231	958,273
cry2Ab	1231	791,297,143
cry2Ac	1219	791,428

Table S4. Length distributions of transcripts and unigenes.

	**Transcript**	**Unigene**
Number of genes	365,596	192,963
Sequences≥500 bp	96,217	44,780
Sequences≥1000 bp	42,403	17,614
N50 value	785	633
N90 value	243	234
Maximum length	31,870	31,870
Minimum length	201	201
Total length	206,813,246	98,811,123
Average length	565.69	512.07

Table S5. Statistical summary of the functional annotations of unigenes in public databases.

Database	Unigenes (percentage)
Total	192,963 (100%)
CDD	21,999 (11.4%)
PFAM	24,787 (12.85%)
KEGG	21,923 (11.36%)
KOG	27,976 (14.50%)
GO	37,946 (19.66%)
NR	67,532 (35.00%)
NT	51,053 (26.46%)
All databases	2,814 (1.46%)

Table S7. Primers for qRT-PCR.

	Gene ID	NR annotation	F primer	R primer
Referenc e genes	TRINITY_DN57 985_c2_g6	ribosomal protein S3	TGATGAATCTCA GCACACCATAGC A	ACGCCTACACGC TCCGAGAT
	TRINITY_DN57 340_c3_g1	EF-1-alpha	CCGCCAACATTA CCACTGAAGTC	ATCCACGACGCA ATTCCTTGAC
qRT-PC R genes	TRINITY_DN71 457_c1_g3	trypsin	CATTGACCCTGT GATCTGCGTAGC	CAGCCGTCGTAG TAGGATGAGAGG
	TRINITY_DN72 624_c0_g1	GST	GATAAACCCTCA GCACTGCGTACC	TGGTCGTCCTTTC CAAACTTGTCTG
	TRINITY_DN64 908_c2_g2	glutathione S-transferase 2-like	CCGACTTACGCTT ATCTGT	TGTAGCCGTTATT CTTTCTG
	TRINITY_DN71 461_c0_g5	carboxyleste rase	TAGTTTTCACTGC GGTTGT	TCATTATGGGTAT TTCTGC
	TRINITY_DN71 660_c2_g3	alkaline phosphatase	ACAAGACAGTCA ACCCACT	TCTGCCTCCTTCT ACAAAA
	TRINITY_DN70 954_c2_g1	cytochrome P450	AGAGGCTTGTCA CGGAATC	CAACTTCGCAGTC GTAAAT
	TRINITY_DN69 684_c2_g3	cytochrome P450	CGATATTGAAGC GATACTGT	TTGGTTGATGATG CCTGTT
	TRINITY_DN56 609_c0_g1	cytochrome P450	CCTGCTCTACTTG TCCGAGAC	AAGCCTTCTTTGC TAAACTCA
